# Selective electrocatalytic denitrification to N_2_ via dual single-atomic sites on double-shelled mesoporous carbon spheres

**DOI:** 10.1016/j.eehl.2025.100172

**Published:** 2025-07-23

**Authors:** Wanchao Song, Mengxuan Wang, Hua Zou, Guoshuai Liu

**Affiliations:** Jiangsu Key Laboratory of Anaerobic Biotechnology, School of Environment & Ecology, Jiangnan University, Wuxi 214122, China

**Keywords:** Electrocatalytic denitrification, N_2_ selectivity, Dual single-atomic catalysts, Mesoporous carbon spheres, N–N coupling, Solid base

## Abstract

Electrocatalytic denitrification (ECDN) offers a sustainable prospect by enabling efficient NO_3_^−^ conversion to harmless N_2_. However, the N_2_-selective ECDN remains challenging due to the sluggish kinetics of N–N coupling during NO_3_^−^ reduction. Here, we developed a novel electrocatalyst of dual single-atomic sites on double-shelled mesoporous carbon spheres (FeNC@MgNC-DMCS) using a continuous sequential modular assembly and pyrolysis approach. The outer Mg–N_4_ shell creates medium basicity sites that function as the proton fence, which optimizes the spatial distribution of H∗ species and suppresses ∗N protonation pathways that would otherwise lead to ammonia formation. Concurrently, the inner Fe–N_4_ shell promotes N–N coupling for N_2_ production. 92.8% NO_3_^−^ removal and 95.2% N_2_ selectivity was achieved by the optimized FeNC@MgNC-DMCS catalyst. Furthermore, long-term flow cell testing demonstrated remarkable durability, highlighting the practical potential of FeNC@MgNC-DMCS for sustainable wastewater treatment applications. This work introduces a catalyst design paradigm that integrates a proton-repelling interface to decouple H∗ availability from N_2_ formation pathways, thereby enabling the development of high-performance ECDN catalysts with balanced activity and selectivity for environmental remediation applications.

## Highlights

• FeNC@MgNC-DMCS achieves 95.2% N_2_ selectivity and 92.8% NO_3_^−^ removal rate via spatial separation of Fe/Mg–N_4_ sites.
• Mg–N_4_ outer shell suppresses H∗ coverage by moderating PCET, enabling Fe–N_4_ inner shell to drive N–N coupling for N_2_ production.
• Scalable modular synthesis ensures long-term stability in flow electrolysis, advancing SACs for sustainable wastewater treatment.

## Graphical abstract

**Figure:**
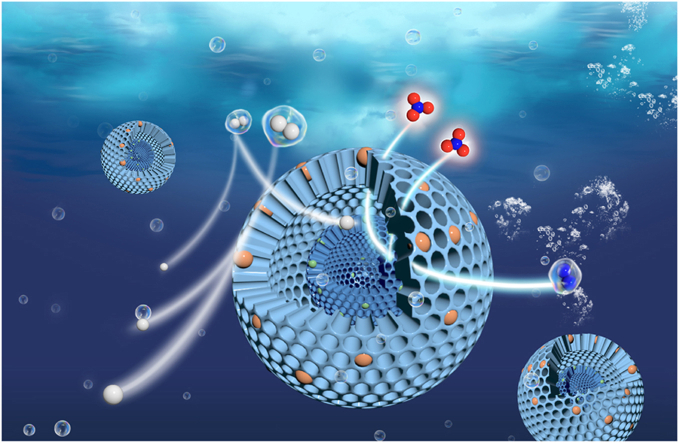

## 1 Introduction

Nitrate (NO_3_^−^) contamination from inadequately treated wastewater is a major environmental and public health concern [[1], [2], [3]]. Various methods have been proposed to address NO_3_^−^ removal, including physicochemical techniques such as membrane separation, adsorption, and conventional biological denitrification [[4], [5], [6]]. Among these, electrocatalytic denitrification (ECDN) processes, which transform NO_3_^−^ into N_2_ gas or NH_3_, have emerged as a promising alternative [7]. Electrochemical processes are particularly appealing for NO_3_^−^ reduction due to their fast reaction rates, eco-friendly nature, and minimal secondary pollution [8,9]. In many cases, electrocatalysts have been designed for ammonia production, aiming to recover resources [[10], [11], [12]]. However, challenges remain in the economic and practical aspects of NH_3_ recovery from wastewater, particularly the complexity and cost involved in separating dilute ammonia from water and its storage [13]. Moreover, NH_3_ poses a higher environmental threat than NO_3_^−^ [14,15]. Therefore, it is highly appreciated to develop an electrocatalyst that can selectively convert NO_3_^−^ to N_2_.

Theoretically, ECDN to N_2_ or NH_3_ proceeds through multiple intermediate steps, with the first 5-electron transfer steps (NO_3_^−^ → NO_2_^−^ → NO → ∗N) being identical for both reaction pathways [16,17]. From the intermediate ∗N, N–N coupling or N–H hydrogenation serves as the key step for N_2_-selective and NH_3_-selective pathways, respectively [18,19]. The primary difference between these two paths arises from the active hydrogen (H∗) produced during the Volmer step (H^+^ ​+ ​e^−^ → H∗) [20,21]. As a potent reducing agent, H∗ reduces adsorbed NO_3_^−^ and nitrogen-containing intermediates [22,23]. Importantly, once ∗N is formed during ECDN, the stepwise hydrogenation pathway (N–H) is kinetically favored over the dimerization route (N–N) (e.g., 2∗N → N_2_), which explains why most catalysts reported in the literature tend to favor NH_3_ production [24,25]. Therefore, theoretically, the N–N coupling reaction can become the dominant pathway by inhibiting the proton-coupled electron transfer (PCET) process associated with N–H hydrogenation and reducing the local coverage of H∗ at the active site.

Solid base catalysts have received significant interest due to their stable deprotonation ability [26,27]. These basic co-catalytic sites act as nucleophiles, attracting H∗ species and preventing the long-range migration of H∗, while simultaneously reducing the local coverage of H∗ at the catalytic site [28,29]. As the H∗ coverage decreases, the ∗N intermediate generated at the catalytic site can more readily interact with another ∗N, promoting N–N coupling for N_2_ production. Notably, single-atom catalysts (SACs) with solid bases, which feature highly dispersed sites and high basicity, overcome the common issues of leaching and poor repeatability associated with traditional solid bases [[30], [31], [32]]. By placing basicity sites near the ECDN active sites as a “proton fence”, this approach rationally controls ion migration and optimizes the local distribution of NO_3_^−^ and H∗ [33,34], representing a promising strategy for achieving ECDN with high N_2_ selectivity. It is essential to note that the deoxidation process in ECDN still requires proton involvement in the initial 5-electron transfer process [35,36], and thus, the basicity should be maintained at a moderate level. Therefore, for N_2_-selective ECDN, it is crucial to design the spatial structure of the regulatory monatomic solid base and catalytic sites carefully.

Based on the context outlined above, we developed a simple yet robust continuous sequential modular assembly and pyrolysis route to produce double-shelled mesoporous carbon spheres (DMCS) with a separated microenvironment of dual single-atomic sites (Fe–N_4_ site inside and Mg–N_4_ site outside) for enhanced catalytic N_2_-selective ECDN, which we denoted as FeNC@MgNC-DMCS. Notably, the Fe–N_4_ sites located in the inner sphere of DMCS can be used as an effective catalyst for N_2_-selective ECDN. The introduction of Mg–N_4_ sites provided a medium basicity site inside the nanospheres, hindering the excessive protonation process of ∗N by the proton fence effect. Without affecting the NO_3_^−^ removal rate (92.8%), the optimized FeNC@MgNC-DMCS manifested a high N_2_ selectivity (95.2%), which was superior to the single-shell mesoporous carbon spheres (MCS) loaded with Fe–N_4_ (27.5%). *In situ* experimental results demonstrated that the basicity Mg–N_4_ sites on the outside of DMCS could act as the proton trap to promote N–N coupling for N_2_ production on Fe–N_4_ site. Subsequently, FeNC@MgNC-DMCS exhibited robust durability in flow electrolysis, indicating significant potential for practical applications. This work not only advances NO_3_^−^ wastewater treatment but also provides novel insights into the application of SACs for environmental remediation.

## 2 Methods

### 2.1 Synthesis of FeNC-MCS

For the synthesis of MCS, 2.5 ​mL ammonia solution and 0.8 ​mL formaldehyde were added to Solution A, which contained 65 ​mL ethanol and 15 ​mL deionized (DI) water. Subsequently, 0.6 ​g resorcinol and 6 ​mL tetrapropoxysilane (TPOS) were added, and the resulting mixture underwent continuous agitation for 24 ​h. The precursors were achieved via centrifugation, followed by drying at 60 ​°C. These precursors were then subjected to thermal treatment under Ar at 800 ​°C for 2 ​h, employing a heating rate of 5 ​°C/min. SiO_2_ template of products was eliminated by etching with 10 ​wt% hydrofluoric acid solution (HF). MCS were harvested by centrifugation, rinsed alternately with DI water multiple times, and finally vacuum-dried at 60 ​°C overnight prior to further utilization.

Solution B was formulated by dispersing 10.6 ​mg Fe(acac)_3_ in 30 ​mL dimethylformamidex (DMF). Solution C was prepared by dispersing 30 ​mg of synthesized MCS in 30 ​mL DMF. Following sonication for 30 ​min, solutions B and C were mixed and magnetically stirred for 24 ​h. The recovered composite powder was calcined in an argon environment at 800 ​°C for 2 ​h, with a programmed temperature increase of 5 ​°C/min. After centrifugation and drying overnight, the final products were isolated and named FeNC-MCS.

### 2.2 Synthesis of FeNC@MgNC-DMCS

FeNC@MgNC-DMCS was synthesized by regrowing carbon spheres using FeNC-MCS as seeds. The procedure was as follows: 2 ​mL of NH_3_·H_2_O and 0.3 ​mL of formaldehyde, and 0.2 ​g of resorcinol were added to 80 ​mL of ethanol-DI water (ethanol: 65 ​mL, DI water: 15 ​mL) dispersion of 20 ​mg FeNC-MCS. Then, 3 ​mL TPOS was introduced into the reaction mixture, which underwent continuous agitation for 24 ​h. The resulting solids were isolated via centrifugation and dried at 60 ​°C. These products were then calcined under argon flow at 800 ​°C for 2 ​h (5 ​°C/min ramp rate), followed by silica template elimination through etching with 10 ​wt% HF. The FeNC-DMCS were recovered by centrifugation, subjected to multiple washing cycles with deionized water and ethanol, and finally vacuum-dried overnight at 60 ​°C. Subsequently, MgPc was loaded onto FeNC-DMCS by the impregnation-pyrolysis process. According to different addition amounts of MgPc (5, 10, and 15 ​mg), the samples obtained were defined as FeNC@MgNC(0.1)-DMCS, FeNC@MgNC(0.2)-DMCS, and FeNC@MgNC(0.3)-DMCS. FeNC@MgNC(0.2)-DMCS was selected as the optimal sample. For the synthesis of MgNC-DMCS, DMCS precursors were prepared by the method of Mg^2+^ impregnation-pyrolysis, and final products were collected by centrifugation and dried overnight.

### 2.3 Electrochemical measurements

Electrochemical measurements were performed using a CHI 760E instrument equipped with a standard three-electrode cell system in 0.05 ​M Na_2_SO_4_ as the electrolyte. A platinum electrode (2.5 ​mm ​× ​2.5 ​mm) and a saturated calomel electrode (SCE) served as the counter electrode and reference electrode, respectively. The catalyst ink used for the working electrode was prepared as follows: 10 ​mg of the catalyst and 80 ​μL of a 5% Nafion solution were added to 1 ​mL of ethanol and sonicated for 1 ​h to ensure thorough mixing. The resulting ink was then drop-coated onto a piece of Ni foam (2.5 ​mm ​× ​2.5 ​mm, loading: 0.2 ​mg/cm^2^). The working electrode was air-dried under ambient conditions before electrochemical testing. The electrocatalytic removal of NO_3_^−^ was conducted in a 50 ​mL H-cell operated in constant voltage mode. Further details regarding the methods can be found in the supplemental information.

## 3 Results and discussion

### 3.1 Synthesis and characterization by FeNC@MgNC-DMCS

**Fig. 1:**
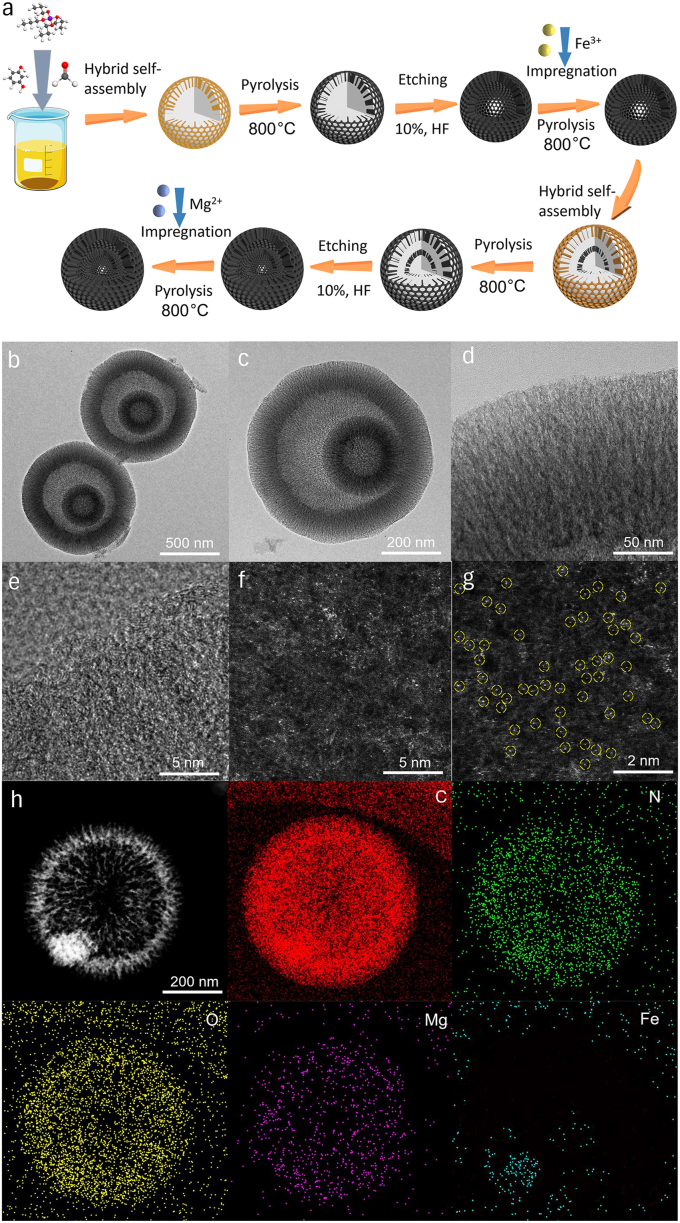
(a) Schematic diagram of sequential synthetic process of FeNC@MgNC-DMCS; (b–e) HRTEM images of the as-obtained FeNC@MgNC-DMCS; (f) HAADF-STEM image of FeNC@MgNC-DMCS; (g) Zoomed-in HAADF-STEM image indicates the dispersion of metal atom sites where bright dots were marked with yellow dashed circles; (h) HAADF-STEM image and EDS mapping of C, N, O, Mg, and Fe elements over FeNC@MgNC-DMCS.

A modular assembly method for preparing double-shelled mesoporous carbon spheres loaded with Fe/Mg single atoms was developed (Fig. 1a). This method began with the preparation of hollow mesoporous carbon spheres through the hybrid co-assembly of phenolic resin and silica (SiO_2_) [37,38]. Under the catalysis of ammonia, the phenolic resin produced by the polymerization of resorcinol and formaldehyde self-assembled with SiO_2_ (via TPOS hydrolysis) in solution to yield phenolic resin/SiO_2_ hybrid spheres [39,40]. After pyrolyzing these hybrid spheres in an argon atmosphere and removing the SiO_2_ with hydrofluoric acid, MCSs with an average shell thickness of about 150 ​nm and a hollow radius of about 125 ​nm were obtained (Fig. S1). Subsequently, FeNC-MCS loaded with Fe atoms were prepared by impregnating the MCSs with a DMF solution of dissolved Fe(acac)_3_ [41], followed by heat treatment and pickling in an argon atmosphere. Analysis of transmission electron microscope (TEM) images (Fig. S2a–d) revealed that Fe loading did not alter the catalyst's morphology and that no metal fringes were observed. Moreover, Fig. S2e and the high-angle annular dark-field scanning transmission electron microscopy (HAADF-STEM) image in Fig. S2f confirmed the presence of a single Fe atom (marked with yellow circles). The corresponding energy-dispersive X-ray spectroscopy (EDS) element mapping image demonstrated that C, N, O, and Fe coexisted and were evenly distributed on the MCS (Fig. S2g).

**Fig. 2:**
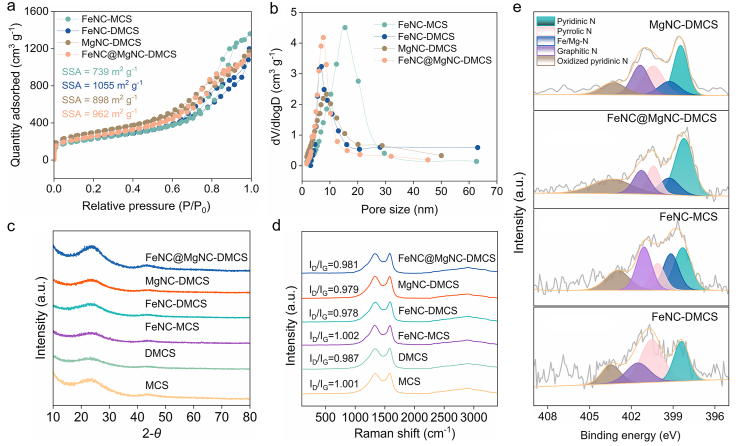
(a) Nitrogen adsorption–desorption isotherms, and (b) pore size distribution curves of FeNC@MgNC-DMCS, MgNC-DMCS, FeNC-MCS, and FeNC-DMCS; (c) XRD patterns and (d) Raman spectra of FeNC@MgNC-DMCS, MgNC-DMCS, FeNC-MCS, FeNC-DMCS, DMCS, and MCS; (e) High-resolution XPS spectra of N 1s for FeNC@MgNC-DMCS, MgNC-DMCS, FeNC-MCS, and FeNC-DMCS.

Subsequently, to obtain the double-shelled structure, we grew another layer of phenolic resin/SiO_2_ on the surface of FeNC-MCS, followed by a pyrolytic-pickling process. The resulting FeNC-DMCS was then impregnated with MgPc in a DMF solution, and an orderly distribution of metal atoms in FeNC@MgNC-DMCS was achieved after a subsequent pyrolysis procedure. The content of the secondary self-assembled phenolic resin/SiO_2_ was optimized. At low concentrations, a complete shell could not be formed (Fig. S3), whereas at high concentrations, a uniform double-shell structure did not develop (Fig. S4). When the content of the secondary precursors was appropriate, uniform double-shelled mesoporous carbon spheres were obtained, featuring an average shell thickness of approximately 260 ​nm and a hollow radius of approximately 435 ​nm (Fig. S5, Fig. 1b and c). Additionally, high-resolution transmission electron microscopy (HRTEM) images indicated that no obvious aggregated metal particles existed within the carbon structure of FeNC@MgNC-DMCS (Fig. 1d and e). Fig. 1f and g presented HAADF-STEM images of the FeNC@MgNC-DMCS shell, which illustrated atomic-sized bright spots. Upon zooming in, most Mg single atoms (marked with yellow circles) were observed. The corresponding EDS element mapping image demonstrated that C, N, O, Mg, and Fe were evenly distributed on the DMCS (Fig. 1h), with no significant metal aggregation or particle formation. Finally, the spatial segregation of Mg and Fe species was unambiguously visualized through EDS elemental mapping. Notably, Fe species were predominantly localized within the inner carbonaceous layer, whereas Mg species exhibited exclusive occupancy in the outer shell structure. This spatial distribution indicated that the outer shell effectively prevented the migration or aggregation of Mg/Fe atoms during pyrolysis [42]. The N_2_ adsorption–desorption isotherms (Fig. 2a and b, Fig. S6) confirmed the presence of highly mesoporous structures [43,44]. The Brunauer–Emmett–Teller (BET) specific surface areas of the synthesized catalysts exhibited significant variations, with 898, 962, 1055, 739, 1109, and 1134 ​m^2^/g for FeNC@MgNC-DMCS, MgNC-DMCS, FeNC-DMCS, FeNC-MCS, DMCS, and MCS, respectively (Fig. 2a and Fig. S6a). Moreover, the Barrett–Joyner–Halenda (BJH) method revealed that the mesopore size distribution of the samples was identical in the range of 7–11 ​nm (Fig. 2b and Fig. S6b). The X-ray diffraction (XRD) analysis showed exclusively two diffuse peaks at 23.0° and 43.5° ​(Fig. 2c), which were attributed to the (002) and (100) planes of disordered graphitic carbon [45]. Similarly, the Raman spectra exhibited comparable D and G band intensity ratios for every sample ​(Fig. 2d), demonstrating characteristic amorphous carbon signatures with comparable disorder [46].

To characterize elemental composition and local coordination, X-ray photoelectron spectroscopy (XPS) and X-ray absorption spectroscopy (XAS) measurements were performed. XPS analysis confirmed exclusively isolated Fe/Mg atoms in FeNC@MgNC-DMCS, MgNC-DMCS, FeNC-DMCS, and FeNC-MCS, with no detectable crystalline metal phases or related compounds (Fig. S7). Spectral fitting of the C 1s region ​(Fig. S8) identified three carbon species: C–C (284.4 ​eV), C–O (285.4 ​eV), and O–C=O (289.0 ​eV), while the O 1s spectrum was characterized by contributions from C=O (532.4 ​eV) and C–O (533.9 ​eV) bonds [47,48]. Additionally, the N 1s spectrum (Fig. 2e) was deconvoluted into peaks corresponding to pyridinic N (398.4 ​eV), pyrrolic N (400.3 ​eV), graphitic N (401.4 ​eV), oxidized pyridinic N (403.3 ​eV), and Fe–N/Mg–N (399.2 ​eV) [49]. The Mg 1s spectrum of MgNC was deconvoluted into a peak centered at 1304.3 ​eV ​(Fig. S9), which was assigned to Mg–N [50]. Furthermore, as shown in Fig. S10, the Fe 2p spectrum indicated the coexistence of Fe^2+^ and Fe^3+^, with Fe^2+^ peaks at 711.1 and 724.2 ​eV and Fe^3+^ peaks at 714.7 and 727.1 ​eV [51,52]. Finally, the Fe atom contents in FeNC@MgNC-DMCS, FeNC-DMCS, and FeNC-MCS, quantified by ICP-MS, were 0.43 ​wt%, 0.42 ​wt%, and 3.16 ​wt%, respectively, while the Mg atom contents in FeNC@MgNC-DMCS and MgNC-DMCS were 0.97 ​wt% and 1.32 ​wt%, respectively (Table S1).

**Fig. 3:**
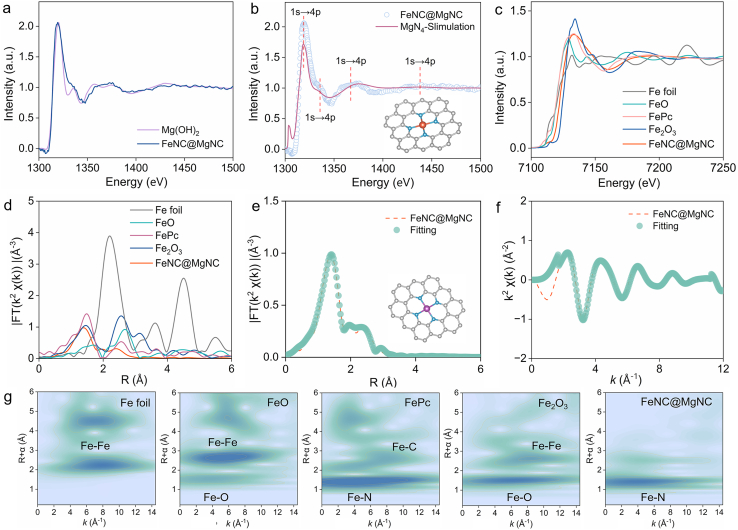
(a) Mg K-edge XANES spectra of FeNC@MgNC-DMCS and Mg(OH)_2_; (b) Experimental and theoretical Mg K-edge XANES spectra of FeNC@MgNC-DMCS (the inset shows the structural model of the Mg–N_4_); (c) Fe K-edge XANES spectra and (d) Fourier-transformed EXAFS spectra for the Fe K-edge of FeNC@MgNC-DMCS, Fe foil, FeO, FePc and Fe_2_O_3_; (e) EXAFS fitting of FeNC@MgNC-DMCS in the R space at the Fe K-edge (the inset shows the structural model of the Fe–N_4_); (f) EXAFS fitting of FeNC@MgNC-DMCS in the K space at the Fe K-edge; (g) Wavelet-transformed EXAFS spectra of Fe foil, FeO, FePc, Fe_2_O_3_, and ​FeNC@MgNC-DMCS from the Fe K-edge EXAFS.

Then, the valence state and coordination environment of iron in FeNC@MgNC-DMCS were further characterized. The Mg K-edge X-ray absorption near-edge structure (XANES) spectra of FeNC@MgNC-DMCS and Mg(OH)_2_ were recorded (Fig. 3a). The near-edge absorption profile of Mg–N–C closely mirrored that of Mg(OH)_2_, which indicated that the valence state of Mg was close to the oxidation state (+2). Moreover, the K-edge resonance (about 1350 ​eV) caused by the 1s→p electron transition is negatively correlated with the coordination number (CN): lower CN value leads to a transfer towards reduced energy [[53], [54], [55]]. As illustrated in Fig. 3b, because the edge of Mg–N–C was left-shifted compared to that of Mg(OH)_2_ (CN ​= ​6), the CN in FeNC@MgNC-DMCS was inferred to be less than 6. Despite XANES sensitivity to oxidation states and coordination environments, it could not confirm the coordination number of the absorbed atoms. As a light element, Mg provided insufficient extended-edge oscillations for Fourier transformation and EXAFS calculations [31,56]. Therefore, the Mg atomic structure in FeNC@MgNC-DMCS was evaluated by comparing the experimental spectra with simulated XANES spectra (Fig. 3b). The 1s-to-empty p state transitions of the Mg–N_4_ structure were consistent with those observed in FeNC@MgNC-DMCS, indicating that the Mg–N_4_ coordination macrocyclic structure was retained in the final product after the pyrolysis of MgPc.

Fig. 3c revealed the Fe K-edge XANES spectra of FeNC@MgNC-DMCS, Fe foil, FeO, FePc, and Fe_2_O_3_. The adsorption threshold position of FeNC@MgNC-DMCS was located between those of FeO and Fe_2_O_3_, indicating that the average metal valence state of Fe in FeNC@MgNC-DMCS lay between them [57]. The Fourier-transformed extended X-ray absorption fine structure (EXAFS) data for FeNC@MgNC-DMCS, Fe foil, FeO, FePc, and Fe_2_O_3_ were illustrated in Fig. 3d. An intense peak at 1.40 ​Å confirmed the existence of a Fe–N scattering path in FeNC@MgNC-DMCS. Quantitative EXAFS fitting (Fig. 3e–f, ​Figs. S11 and S12) ​was performed to authenticate the local coordination configuration of Fe in FeNC@MgNC-DMCS [5]. The fitting results revealed that each Fe atom in FeNC@MgNC-DMCS was coordinated with four N atoms at 2.01 ​Å (Table S2). In addition, the Fe K-edge wavelet transform (WT)-EXAFS of FeNC@MgNC-DMCS exhibited a significant peak at 4.5 ​Å^−1^, closely resembling that observed in FePc and identified as the Fe–N bond (Fig. 3g), which further demonstrated the isolated characteristics of the Fe species [58]. This finding was distinct from the characteristic Fe–Fe bonds observed at 6.8 ​Å^−1^, 5.9 ​Å^−1^, and 8.7 ​Å^−1^ in Fe foil, FeO, and Fe_2_O_3_, respectively.

### 3.2 ECDN performance by FeNC@MgNC-DMCS

**Fig. 4:**
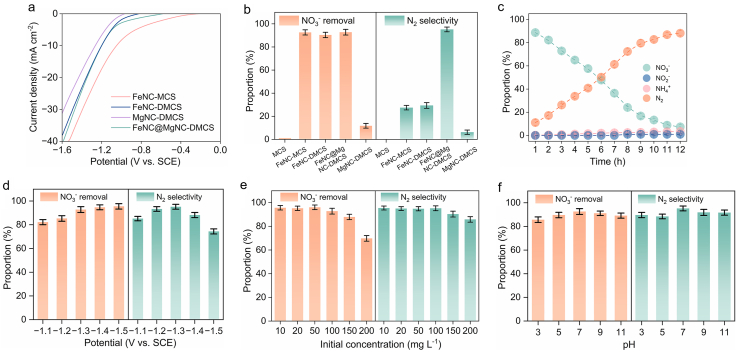
(a) LSV curves of FeNC-MCS, FeNC-DMCS, MgNC-DMCS, and FeNC@MgNC-DMCS in an electrolyte of 0.05 ​M Na_2_SO_4_ with 100 ​mg/L NO_3_^−^; (b) NO_3_^−^ removal rate and N_2_ selectivity of MCS, FeNC-MCS, FeNC-DMCS, MgNC-DMCS, and FeNC@MgNC-DMCS for 12 ​h; (c) Concentration of NO_3_^−^, NO_2_^−^, NH_4_^+^, and N_2_ as a function of time during the NO_3_^−^ reduction over FeNC@MgNC-DMCS; ECDN activity over FeNC@MgNC-DMCS at different (d) cathode potential, (e) initial pH values, and (f) initial concentrations of NO_3_^−^.

The ECDN performance of FeNC@MgNC-DMCS was evaluated in a 50 ​mL dual-chamber electrolytic cell under ambient conditions (Fig. S13). The reactants and reduction products, including NO_3_^−^, NO_2_^−^, and NH_4_^+^, were detected using UV-vis spectrophotometry (Fig. S14). Initially, we compared the electrochemical performance of FeNC@MgNC-DMCS electrocatalysts with various Mg atomic contents (Fig. S15). FeNC@MgNC(0.1)-DMCS, FeNC@MgNC(0.2)-DMCS, and FeNC@MgNC(0.3)-DMCS revealed NO_3_^−^ removal rates of 93.7%, 92.8%, and 74.4% and N_2_ selectivities of 68.6%, 95.2%, and 94.9%, respectively. These electrocatalytic results clearly indicated that an optimized MgPc addition amount (0.2 ​mmol) provided the best catalytic effect for N–N coupling during the ECDN process. The linear sweep voltammograms (LSV) for the ECDN were presented in Fig. 4a and Fig. S16. The FeNC@MgNC-DMCS revealed an onset potential of 0.52 ​V vs. SCE, between FeNC-DMCS (−0.78 ​V vs. SCE) and FeNC-MCS (−0.22 ​V vs. SCE), and much more positive than that of MgNC-DMCS (0.95 ​V vs. SCE). This could be that Mg single atom serves as a proton barrier, effectively impeding local proton/active hydrogen migration, thereby lowering the onset potential of nitrate reduction [59,60]. The presence of NO_3_^−^ in electrolyte induces a substantial increase in current density for FeNC@MgNC-DMCS electrodes in comparison to blank Na_2_SO_4_ electrolytes (Figs. S17 and S18), indicating the ECDN reaction was initialized by FeNC@MgNC-DMCS. Furthermore, electrochemical impedance spectroscopy (EIS) evidenced that FeNC@MgNC-DMCS possessed a moderate Nyquist graph diameter, consistent with the LSV results, which meant that its charge transfer capacity lay between that of FeNC-MCS and MgNC-DMCS (Fig. S19). Chronoamperometry measurements were conducted for 12 ​h in a 0.05 ​M Na_2_SO_4_ solution containing 100 ​mg/L NO_3_^−^ (Fig. 3b). The ECDN performances of MCS and MgNC-DMCS were also tested and found to be negligible, with NO_3_^−^ removal rates of only 1.1% and 11.8%, respectively. While FeNC-MCS, FeNC-DMCS, and FeNC@MgNC-DMCS exhibited similar NO_3_^−^ removal rate (92.6%, 90.4%, and 92.8%, respectively), their N_2_ selectivity varied significantly (27.5%, 29.3%, and 95.2%, respectively; Fig. 4b and c), implying that the FeNC@MgNC-DMCS with double-shelled structure was favorable for N_2_-selective ECDN by proton fence effect. To further verify this, we tested the ECDN activity of FeMg dual-atom catalyst with a single-shelled structure (Fig. S20). FeMg-MCS exhibited N_2_ selectivity of 82.4%; however, its NO_3_^−^ removal rate was only 61.2% of that achieved by FeMg-MCS. Additionally, it showed a higher NO_2_^−^ accumulation (about 7%) and significantly lower ECDN activity compared to FeNC@MgNC-DMCS. This may be attributed to the relatively close distance between Fe and Mg atoms, which leads to excessive proton interception by the Mg–N_4_ site, thereby hindering the NO_3_^−^ reduction kinetics. These results further confirm that the enhanced ECDN performance of FeNC@MgNC-DMCS arises from the moderate proton interception capacity of the outer Mg–N_4_ shell.

Subsequently, electrolysis tests were performed under various applied potentials from −1.1 ​V to −1.5 ​V vs. SCE to investigate the ECDN performance of FeNC@MgNC-DMCS (Fig. 4d). The ECDN performance exhibited a positive correlation with decreasing reduction potential, stabilizing within a plateau at −1.3 ​V vs. SCE. This voltage represented the optimal operational point where nitrate reduction achieved enough energy with minimal H_2_ evolution reaction. Meanwhile, exceeding −1.3 ​V vs. SCE induced a marked decline in N_2_ selectivity (74.4% N_2_ selectivity at −1.5 ​V vs. SCE), probably owing to increased competition from the H_2_ evolution reaction [[61], [62], [63]]. In addition, the ECDN tests performed at different initial pH values (3−11) all indicated high NO_3_^−^ removal rate (85.7%–92.6%) and N_2_ selectivity (89.6%–95.2%) (Fig. 4e). These results confirmed that the N_2_-ECDN performance of FeNC@MgNC-DMCS was robust over a wide range of initial pH. Moreover, the excellent N_2_ selectivity of FeNC@MgNC-DMCS was maintained even when the initial NO_3_^−^ concentration varied from 10 ​mg/L to 200 ​mg/L, as shown in Fig. 4f. However, under high-concentration conditions, the NO_3_^−^ removal rate began to decline, reaching 69.8% at 200 ​mg/L. Furthermore, the ECDN performance of FeNC@MgNC-DMCS was systematically evaluated under interference from common coexisting ions (Cl^−^, CO_3_^2−^) and humic acid (HA) (Fig. S21). The results revealed that the catalyst maintained stable catalytic performance across the coexistence of Cl^−^, CO_3_^2−^, and HA, achieving NO_3_^−^ removal rate exceeding 90% and N_2_ selectivity above 93%. Therefore, FeNC@MgNC-DMCS exhibited robust anti-interference capability in complex aqueous matrices. Notably, the ECDN performance of FeNC@MgNC-DMCS catalysts was superior to that reported in most previous studies in terms of N_2_ selectivity (Table S3).

### 3.3 ECDN reduction mechanism and pathway by FeNC@MgNC-DMCS

**Fig. 5:**
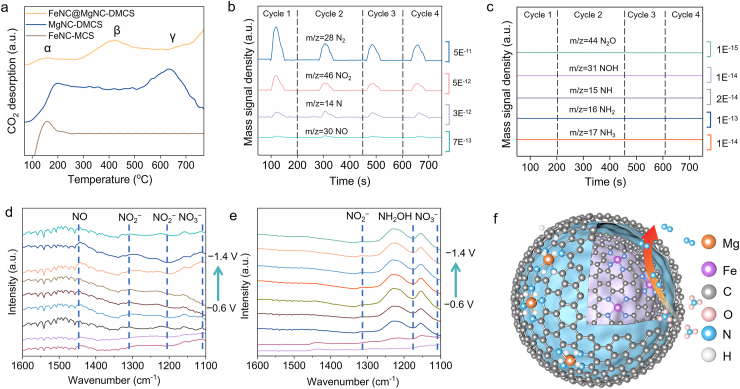
(a) CO_2_-TPD profiles of FeNC-MCS, MgNC-DMCS, and FeNC@MgNC-DMCS; (b, c) DEMS analysis of nitrogen species during the NO_3_^−^ reduction on FeNC@MgNC-DMCS; *In situ* IR spectra while ramping down the potential from −0.6 ​V to −1.4 ​V vs. SCE on (d) FeNC@MgNC-DMCS and (e) FeNC-MCS; (f) Mechanism diagram showing the ECDN over FeNC@MgNC-DMCS.

The basicity of the prepared electrocatalysts was investigated by temperature-programmed desorption of CO_2_ (CO_2_-TPD). The desorption peaks of CO_2_ can be tentatively divided into three parts named α, β, and γ, located at about 150, 450, and 630 ​°C, respectively (Fig. 5a). All materials exhibited the α-peak, which was assigned to mesostructure-physisorbed CO_2_. FeNC-MCS uniquely failed to exhibit β/γ desorption above 150 ​°C. In contrast, MgNC-DMCS revealed a γ peak in addition to the physically adsorbed α peak. According to previous reports, the CO_2_ desorption at 630 ​°C is exceeded that of typical solid superbases such as K_2_O/Al_2_O_3_ (600 ​°C), CaO/Al_2_O_3_ (580 ​°C), and K_2_O/Ba-MCM-41 (590 ​°C) [30,64], thus Mg–N_4_ in MgNC-DMCS exhibited a high basic sites property [31,33]. It was further noted that the β peak appeared at 450 ​°C for FeNC@MgNC-DMCS, while the γ peak exhibited only a weak signal. This observation was likely caused by the lower Mg atom content in FeNC@MgNC-DMCS compared to MgNC-DMCS, and the CO_2_ desorption at this temperature corresponded to medium basicity [30,64]. Generally, transition metal-based SACs are not considered to possess basicity characteristics [65], and medium basicity FeNC@MgNC-DMCS was prepared by controlling the adsorption amount of MgPc in this study.

To validate the interference of Mg–N_4_ sites on the PCET process, the formation of H during the ECDN was examined using different catalyst cathodes in electron paramagnetic resonance (EPR) spectra experiments with the radical trapping reagent 5,5-dimethyl-1-pyrroline-N-oxide (DMPO). For FeNC-MCS and FeNC-DMCS, a gradual decrease in Fe atom content corresponded to a reduction in the intensity of the DMPO-H characteristic peak (Fig. S22), indicating the formation of H∗ during electrolysis. This was a factor that may have contributed to the poor N_2_ selectivity of the isolated Fe site. In contrast, the DMPO-H characteristic peak was not detected in MgNC-DMCS and FeNC@MgNC-DMCS, suggesting that Mg sites affected the transformation of H, thereby confirming the conclusion presented in Fig. 4a. Additionally, the Faradaic efficiency (FE) was calculated to assess the degree of interference by Mg sites on the ECDN process (Fig. S23). On the other hand, as the potential decreased from −1.1 to −1.5 ​V vs. SCE, FE decreased from 54.3% to 35.7%, reaching its highest value (59.2%) at −1.3 ​V vs. SCE. These results indicated that although Mg sites interfered with the PCET process, the Fe site within DMCS was still able to complete the ECDN process relatively efficiently.

Subsequently, *in situ* differential electrochemical mass spectrometry (DEMS) was performed for high-sensitivity online detection of evolving gas species during cathodic processes [4]. Molecular identification via *m*/*z* signatures coupled with continuous quantification of intermediates/products was achieved. As indicated in Fig. 5b, the *m*/*z* 28 signal, attributed to N_2_, was the strongest, confirming the high N_2_ selectivity of FeNC@MgNC-DMCS. During the *in situ* tests, the *m*/*z* signals of 46, 14, and 30 in the DEMS curves of FeNC@MgNC-DMCS corresponded to ∗NO_2_, ∗N, and ∗NO in four continuous cycles, respectively. The signal intensities of these intermediates were several orders of magnitude smaller than that of N_2_, indicating that N_2_ was the main gaseous product. Conversely, the *m*/*z* signals associated with 31 (∗NOH), 15 (∗NH), 16 (∗NH_2_), and 17 (NH_3_) did not exhibit significant peaks, indicating that FeNC@MgNC-DMCS produced no ammonia during the ECDN (Fig. 5c), and these DEMS results agreed with the UV-vis findings. In particular, the *m*/*z* 44 signal (∗N_2_O) was negligible, while the *m*/*z* 14 signal (∗N) was more prominent, suggesting that FeNC@MgNC-DMCS catalyzed the N–N coupling process by combining two N atoms rather than two NO molecules [17]. In addition to online DEMS, *in situ* infrared (IR) spectroscopy was also employed to investigate the reaction mechanism (Fig. 5d and e). Absorption bands at 1203 and 1305 ​cm^−1^ were associated with NO_2_^−^, with the highest intensity signal observed at a cathodic potential of −1.3 ​V vs. SCE. As the potential increased, the emergence of a characteristic band at 1477 ​cm^−1^ (assigned to NO) at −0.9 ​V vs. SCE suggested the step-wise reduction of NO_2_^−^ to NO (Fig. 5d). Furthermore, an absorption band at 1176 ​cm^−1^ was assigned to the ∗NH_2_OH intermediate (Fig. 5e), which exclusively appeared in the infrared spectrum of FeNC-MCS [66]. Notably, the stretching vibration of ∗NH_2_OH was absent in the IR spectra of FeNC@MgNC-DMCS, confirming that FeNC@MgNC-DMCS highly selective converted NO_3_^−^ to N_2_. For the ECDN process, the proton in water or the H∗ produced by water dissociation could be tapped by the basicity of Mg–N_4_ in the outer shell of FeNC@MgNC-DMCS [29,36,63], and then the surface coverage of H∗ or proton was insufficient to drive the hydrogenation pathway (N–H) of ECDN. Consequently, the ∗N intermediate would be electrochemical coupling to form N_2_ (2∗N → N_2_) of Fe–N_4_ on the inner shell. In other words, the Mg–N_4_ in the outer shell functioned as the proton fence to balance the trade-off of hydrogenation or coupling reaction of ∗N, which is the branching reaction pathway for the ECDN process. Based on these findings, the most likely ECDN pathway for FeNC@MgNC-DMCS was identified, as shown in Eq. (1), (2), (3), (4), (5), (6), and a schematic diagram of the ECDN catalytic mechanism for FeNC@MgNC-DMCS was presented in Fig. 5f.

(1) $${{\text{NO}}_{3}^{-}}_{\left(\text{aq}\right)}\;\to \;{{\text{NO}}_{3}^{-}}_{\left(\text{ad}\right)}$$

(2) $${{\text{NO}}_{3}^{-}}_{\left(\text{ad}\right)}+2H^{+}+2e^{-}\;\to \;{{\text{NO}}_{2}^{-}}_{\left(\text{ad}\right)}+H_{2}O$$

(3) $${{\text{NO}}_{2}^{-}}_{\left(\text{ad}\right)}+2H^{+}+e^{-}\;\to \;{\text{NO}}_{\left(\text{ad}\right)}+H_{2}O$$

(4) NO(ad)+2H++2e−→N(ad)+H2O

(5) N(ad)+N(ad)→N2(ad)

(6) $$N_{2\;\left(\text{ad}\right)}\;\to \;N_{2\;\left(\text{aq}\right)}$$

### 3.4 Flow cell design and performance of the NO_3_^−^ reduction

**Fig. 6:**
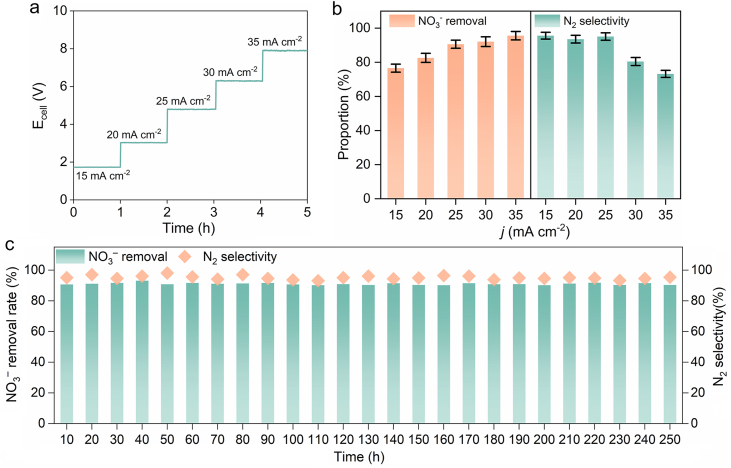
(a) Discharging curves at different current densities; (b) NO_3_^−^ conversion and N_2_ selectivity derived from the flow reactor at different current densities; (c) Stability evaluation of flow reactor for continuous 250 ​h in NO_3_^−^-containing simulant wastewater.

To investigate the practical performance of FeNC@MgNC-DMCS for ECDN, we assembled a well-designed flow cell using a boron-doped diamond (BDD) as the anode and a graphite sheet decorated with FeNC@MgNC-DMCS as the cathode to evaluate the potential application of the catalyst in real wastewater treatment (Fig. S24). Reverse osmosis concentrate (ROC) served as the target wastewater (Table S4), and water was fed into the reactor from the bottom. The continuous stability of the output discharge current density, which increased from 15 to 35 ​mA/cm^2^, suggested that the flow cell possessed outstanding discharge capability with fast mass transfer and good conductivity (Fig. 6a) [46]. As the current density increased from 15 to 25 ​mA/cm^2^, the NO_3_^−^ removal improved from 76.6% to 90.6%, while the N_2_ selectivity remained around 95% (Fig. 6b). However, at a higher current density of 35 ​mA/cm^2^, although the NO_3_^−^ removal rate reached 95%, the N_2_ selectivity dropped to 73.2%. This finding indicated that 25 ​mA/cm^2^ was the optimal operating parameter for the reactor. The long-term test, as illustrated in Fig. 6c, demonstrated that FeNC@MgNC-DMCS exhibited remarkable stability. After 250 ​h at 25 ​mA/cm^2^, neither the nitrate removal rate (>90%) nor the N_2_ selectivity (>93%) changed significantly, indicating superb cycling stability. Moreover, the LSV curves remained almost unchanged for FeNC@MgNC-DMCS before and after the cycling tests (Fig. S25), highlighting its excellent electrochemical activity under long-term operating conditions. The structural stability of FeNC@MgNC-DMCS after the cycling tests was characterized by TEM, which illustrated that the catalyst morphology and element distribution were without obvious change (Fig. S26). Furthermore, XRD and XPS analyses together indicated that FeNC@MgNC-DMCS had the same structural characteristics before and after cycling, further confirming the high stability of the catalyst (Figs. S27 and S28). Finally, considering that leakage of Fe^3+^ and Mg^2+^ during the electrocatalytic process was prone to cause secondary pollution of water bodies, we tested each electrolyte after circulation by ICP-MS. The results illustrated that the leaching amounts of Fe^3+^ and Mg^2+^ were merely 1.58 and 6.17 ​μg/L, respectively, far below the World Health Organization (WHO) maximum permissible level in drinking water (0.2 and 20 ​mg/L, respectively) [67,68], which further indicated that the FeNC@MgNC-DMCS was structurally stable and environmental safety. Based on these experimental results, FeNC@MgNC-DMCS demonstrated excellent chemical structural stability as well as promising durability as efficient catalysts for electrocatalytic ECDN.

## 4 Conclusions

We designed a dual single-atomic catalyst with a double-shell mesoporous carbon sphere structure to enhance the selective reduction of NO_3_^−^ to N_2_. Our findings indicate that the Mg atoms in the outer shell provide stable medium basicity sites, which effectively hinder the excessive formation of H∗ and inhibit the hydrogenation of N–H during the ECDN process. The Mg-assisted FeNC@MgNC-DMCS exhibited a NO_3_^−^ removal rate of 92.8% and an N_2_ selectivity of 95.2%, with NO_3_^−^ removal levels exceeding 90%. In contrast, Fe-MCS containing isolated Fe–N_4_ sites achieved only 27.5% N_2_ selectivity. Long-term flow cell testing demonstrated significant effectiveness and durability when treating realistic ROC wastewater, highlighting the practical potential of FeNC@MgNC-DMCS for sustainable wastewater treatment.

## CRediT authorship contribution statement

**Wanchao Song:** Formal analysis, Conceptualization, Writing – original draft, Data curation. **Mengxuan Wang:** Data curation, Formal analysis. **Hua Zou:** Validation, Project administration, Supervision. **Guoshuai Liu:** Supervision, Validation, Funding acquisition.

## Declaration of competing interest

The authors declare that they have no known competing financial interests or personal relationships that could have appeared to influence the work reported in this paper.
