# Supplementary material for: Selective electrocatalytic denitrification to N2 via dual single-atomic sites on double-shelled mesoporous carbon spheres
Source: Eco Environ Health. 2025 Jul 23;4(3):100172. doi: 10.1016/j.eehl.2025.100172 (PMC12341511; doi:10.1016/j.eehl.2025.100172)
Supplement: Multimedia component 1 [file mmc1.docx]

Supporting information

Selective electrocatalytic denitrification to N_2_ via dual single-atomic sites on double-shelled mesoporous carbon spheres

Wanchao Song ^a^, Mengxuan Wang ^a^, Hua Zou ^a^*, Guoshuai Liu ^a^*

^a^ Jiangsu Key Laboratory of Anaerobic Biotechnology, School of Environment & Ecology, Jiangnan University, Wuxi 214122, China.

* Corresponding authors

E–mail: zouhua@jiangnan.edu.cn (H. Zou); [guoshuailiu@jiangnan.edu.cn](mailto:guoshuailiu@jiangnan.edu.cn) (G. Liu).

Summary

Number of Pages: Page S1–Page S41

Number of Figures: Fig. S1–Fig. S28

Number of Tables: Table S1–Table S4

1. Supplementary Methods

1.1 Chemicals

Sodium nitrate (NaNO_3_, ≥99%), potassium nitrate (KNO_3_, ≥99%), hydrochloric acid (HCl, 35%), hydrofluoric acid (HF, ≥40%), ammonia, aqua, (NH_3_·H_2_O, 25-28%), formaldehyde (CH_2_O, ≥37%), ethanol (CH_3_CH_2_OH, ≥99.8%), sodium sulfate (Na_2_SO_4_, 99.0%) were purchased from Sinopharm Chemical Reagent Co., Ltd., SCRC, China. Tetrapropyl orthosilicate (TPOS, (C_3_H_7_O)_4_Si, ≥97%), Iron(III) acetylacetonate (Fe(acac)_3_, ≥98%), resorcinol (C_6_H_6_O_2_, ≥99%), N,N-Dimethylformamide (DMF, ≥99.5%),  Magnesium(II) Phthalocyanine (MgPc, C_32_H_16_MgN_8_, ≥95%) were purchased from Aladdin Industrial Inc. (Shanghai, China). All the chemicals were used as received without further purification. Deionized water (DI water) with 18.2 MΩ·cm was used in all working solution and measurements.

1.2 Differential electrochemical mass spectrometry (DEMS) measurements

Differential electrochemical mass spectrometry (DEMS, PM-DEMS) was employed to analyze the intermediates and products formed during the reduction of NO_3_^−^. To prepare the catalyst ink, 5 mg of catalyst powder, 1.0 mL of deionized water, 1.0 mL of ethanol, and 50.0 µL of 5 wt% Nafion solution were combined and sonicated for at least 30 minutes to ensure homogeneity. The resulting ink was then uniformly drop-coated onto a glassy carbon electrode and allowed to dry slowly under ambient conditions. The glassy carbon electrode coated with the catalyst, a platinum wire, and a saturated calomel electrode were used as the working, counter, and reference electrodes, respectively. To maintain an inert atmosphere, argon gas was continuously bubbled into the electrolyte before and during the DEMS measurements. An aqueous solution containing 100 mg L^−1^ NaNO_3_ and 0.1 M Na_2_SO_4_ was introduced into a custom-made electrochemical cell using a peristaltic pump. Linear sweep voltammetry (LSV) was performed from 0 to −1.9 V vs. SCE at a scan rate of 0.01 V·s^−1^ until a steady baseline was observed. Subsequently, the corresponding mass signals were recorded. After each test concluded and the mass signal returned to the baseline, a new cycle was initiated under identical conditions to minimize potential errors in the DEMS measurements. The experiment was terminated after completing four cycles.

1.3 Determination of ion concentration

The concentrations of NO_3_^–^, NO_2_^–^, and NH_4_^+^ were measured using an ultraviolet-visible spectrophotometer (HITACHI U-3900), and the corresponding concentration-absorbance calibration curves are presented in Fig. S16. The specific detection methods [1, 2] are as follows:

Detection of NO_3_^−^

To determine the NO_3_^–^ concentration, a specific volume of electrolyte was extracted from the electrolytic cell and diluted to 50 mL, which fell within the detection range. Subsequently, 1 mL of 0.1 M HCl and 0.1 mL of 0.8 wt% sulfamic acid solution were added to the diluted solution. The absorption spectrum was then recorded using the ultraviolet-visible spectrophotometer, focusing on the absorption intensities at wavelengths of 220 nm and 275 nm. The NO_3_^–^ concentration was calculated using the standard calibration curve, which was generated by measuring the UV-visible spectra of KNO_3_ solutions of known concentrations (prepared by drying KNO_3_ at 105–110 °C for 2 h before use).

Detection of NO_2_^−^

Preparation of color reagent: A solution was prepared by dissolving 4 g of p-aminobenzenesulfonamide and 0.2 g of N-(1-naphthyl)ethylenediamine dihydrochloride in a mixture of 50 mL ultrapure water and 10 mL phosphoric acid. For the detection of NO_2_^–^, a specific volume of electrolyte was extracted from the electrolytic cell and diluted to 50 mL to ensure it was within the detection range. Next, 1 mL of the prepared color reagent was added to the diluted solution and mixed thoroughly. After allowing the solution to stand for 20 min, the absorption spectrum was measured using the ultraviolet-visible spectrophotometer, focusing on the absorption intensity at a wavelength of 540 nm. The NO_2_^–^ concentration was calculated using the standard calibration curve.

Determination of NH_4_^+^

The NH_4_^+^ concentration was determined using Nessler’s reagent method. A specific volume of electrolyte was extracted from the electrolytic cell and diluted to 50 mL to ensure it was within the detection range. Subsequently, 1 mL of potassium sodium tartrate solution and 1 mL of Nessler’s reagent were added to the diluted solution. After allowing the solution to stand for 20 minutes, the absorption spectrum was recorded using the ultraviolet-visible spectrophotometer, focusing on the absorption intensity at a wavelength of 420 nm. The NH_4_^+^ concentration was calculated using the standard calibration curve.

1.4 Calculation of nitrate conversion and product selectivity

The NO_3_^–^ removal rate was calculated:

$$\text{R(}\text{NO}_{\text{3}}^{-}\text{)}\text{ }\text{= }\frac{\text{C}_{\text{0}}\text{(}\text{NO}_{\text{3}}^{-}\text{)}\text{−}\text{C}_{\text{t}}\text{(}\text{NO}_{\text{3}}^{-}\text{)}}{\text{C}_{\text{0}}\text{(}\text{NO}_{\text{3}}^{-}\text{)}}\text{×}\text{100\%}$$

The selectivity of the products was calculated:

$$\text{S(}\text{NO}_{\text{2}}^{-}\text{)}\text{ }\text{= }\frac{\text{C}_{\text{t}}\text{(}\text{NO}_{\text{2}}^{-}\text{)}}{\text{C}_{\text{0}}\text{(}\text{NO}_{\text{3}}^{-}\text{)}\text{−}\text{C}_{\text{t}}\text{(}\text{NO}_{\text{3}}^{-}\text{)}}\text{×}\text{100\%}$$

$$\text{S(}\text{NH}_{\text{4}}^{\text{+}}\text{)}\text{ }\text{= }\frac{\text{C}_{\text{t}}\text{(}\text{NH}_{\text{4}}^{\text{+}}\text{)}}{\text{C}_{\text{0}}\text{(}\text{NO}_{\text{3}}^{-}\text{)}\text{−}\text{C}_{\text{t}}\text{(}\text{NO}_{\text{3}}^{-}\text{)}}\text{×}\text{100\%}$$

$$\text{S(}\text{N}_{\text{2}}\text{)= }\frac{\text{C}_{\text{0}}\text{(}\text{NO}_{\text{3}}^{-}\text{)}\text{−}\text{C}_{\text{t}}\text{(}\text{NO}_{\text{2}}^{-}\text{)}\text{−}\text{C}_{\text{t}}\text{(}\text{NH}_{\text{4}}^{\text{+}}\text{)}}{\text{C}_{\text{0}}\text{(}\text{NO}_{\text{3}}^{-}\text{)}\text{−}\text{C}_{\text{t}}\text{(}\text{NO}_{\text{3}}^{-}\text{)}}\text{×}\text{100\%}$$

where C_0_(NO_3_^−^, mg L^−1^) and C_t_(NO_3_^−^, mg L^−1^) are the initial nitrate (NO_3_^−^) concentration and nitrate concentration after different electrocatalytic reduction time, respectively. C_t_(NO_2_^−^-N) and C_t_(NH_4_^+^-N) represent the concentrations of nitrite and ammoniums.

1.5 Calculation of the Faradaic efficiency (FE) for N_2_ production

$$\text{FE= }\frac{\text{[C}_{\text{0}}\text{(}\text{NO}_{\text{3}}^{-}\text{)}\text{−}\text{C}_{\text{t}}\text{(}\text{NO}_{\text{3}}^{-}\text{)]}\text{×}\text{N}\text{×}\text{F}\text{×}\text{V}\text{×}\text{S(}\text{N}_{\text{2}}\text{)}}{\text{M*Q}}\text{×}\text{100\%}$$

where N is the number of transferred electrons (5 for NO_3_^−^ to N_2_), F is the Faradaic constant (96,485 C mol^−1^), V is the volume of electrolyte (50 mL), M is the molar mass of nitrogen (14 g mol^−1^), Q is the total charge passing the electrode [3].

1.6 *In situ IR spectroscopy* measurements

It was performed with a Thermo-Fisher Nicolet iS20 equipped with a liquid nitrogen-cooled HgCdTe (MCT) detector using a VeeMax III ATR accessory (Pike Technologies). All In situ IR measurements were acquired by averaging 64 scans at a spectral resolution of 4 cm^−1^. 5 mg catalyst powder, 1.0 mL deionized water, 1.0 mL ethanol, and 50.0 μL Nafion solution (5 wt%) were mixed and sonicated for at least 30 min to form a homogeneous ink. Catalyst ink was uniformly drop-coated onto the Ge prism and left to dry slowly. The electrolyte was 1000 ppm NaNO_3_−N and 0.1 M Na_2_SO_4_, which was constantly purged with Ar during the measurements. For electrochemical measurements, the potential was swept between −0.6 and −1.4 V vs. SCE at 2 mV S^−1^. The spectra under open circuit potential (OCP) were recorded for comparison.

1.7 Characterizations

The microstructure of synthesized samples was examined by High-resolution transmission electron microscopy (HRTEM, JEOL JEM-2100). High-angle annular dark-field scanning transmission electron microscopy (HAADF-STEM) and energy-dispersive X-ray spectroscopy (EDS) mapping analyses were conducted using a JEM-ARM300F2 microscope equipped with a spherical aberration correction system. X-ray diffraction (XRD) patterns were recorded using a Bruker D8 ADVANCE Phaser (scan range of 10°–80°). Raman spectroscopy data acquisition was conducted on a Horiba LabRAM HR Evolution spectrometer with a 532 nm laser source. X-ray photoelectron spectroscopy (XPS) data were acquired using Thermo ESCALAB QXi spectrometer. The Mg K-edge soft X-ray absorption spectra (XAS) were acquired at the BL08U-1A beamline at the Shanghai Synchrotron Radiation Facility (SSRF). Additionally, Fe K-edge XAS data were collected at the BL14W1 beamline at SSRF and analyzed using the Athena software package. DMPO spin-trapping electron paramagnetic resonance (EPR) spectra were obtained using a Bruker Magnettech EPR5000 spectrometer in an electrolyte of 0.05 M Na_2_SO_4_ at −1.3 V vs. SCE. The online analysis of electrochemical intermediates/products was enabled by Differential electrochemical mass spectrometry (DEMS, PM-DEMS, Shanghai Jingpro Co., Ltd.) The metal contents of the catalysts, as well as the leaching mass of metal ions, were quantified using inductively coupled plasma mass spectrometry (ICP-MS, Agilent 7850).

1.8 Flow cell assembly and electrochemical test

Fig. S23 provided a schematic diagram and a photograph of flow cell, respectively. In a standard flow cell configuration, graphite sheet decorated with FeNC@MgNC-DMCS and boron-doped diamond (BDD) functioned as the cathode and anode (6.0 cm × 3.0 cm), respectively, loaded within a 200 mL electrolysis cell. The inlet was positioned beneath the reactor, consistently supplying electrolyte into the cell at a rate of 0.2 L min^−1^ via a peristaltic pump. Subsequently, post-electrolysis water is discharged from the top. The electrolyte utilized was a reverse osmosis concentrates (ROC) containing NO_3_^−^ (without Cl^−^), obtained from the wastewater treatment plant of Pengyao Environmental Protection Co., Ltd at Yixing, detailed water quality parameters can be seen in Table S4.

2. Supplementary Figures


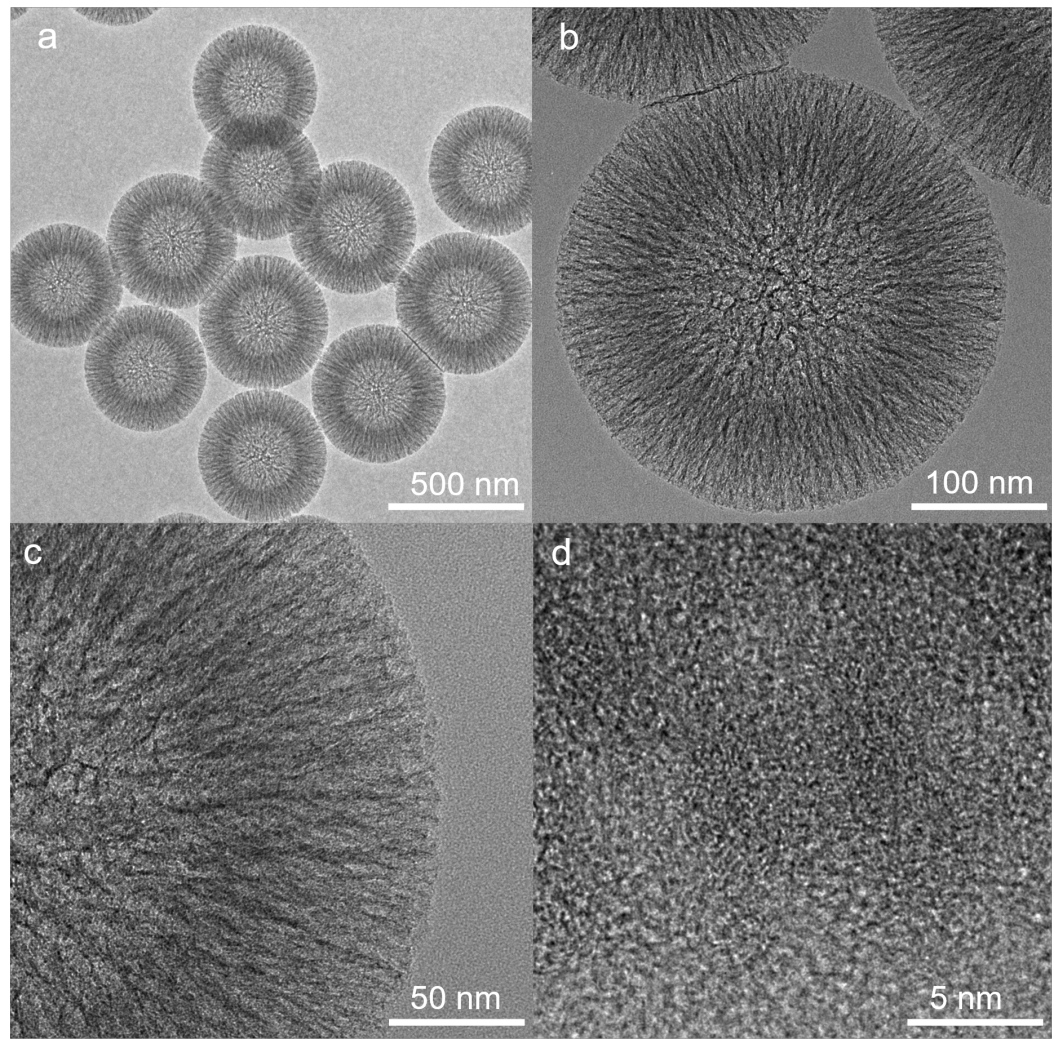


Fig. S1. HRTEM images of MCS.


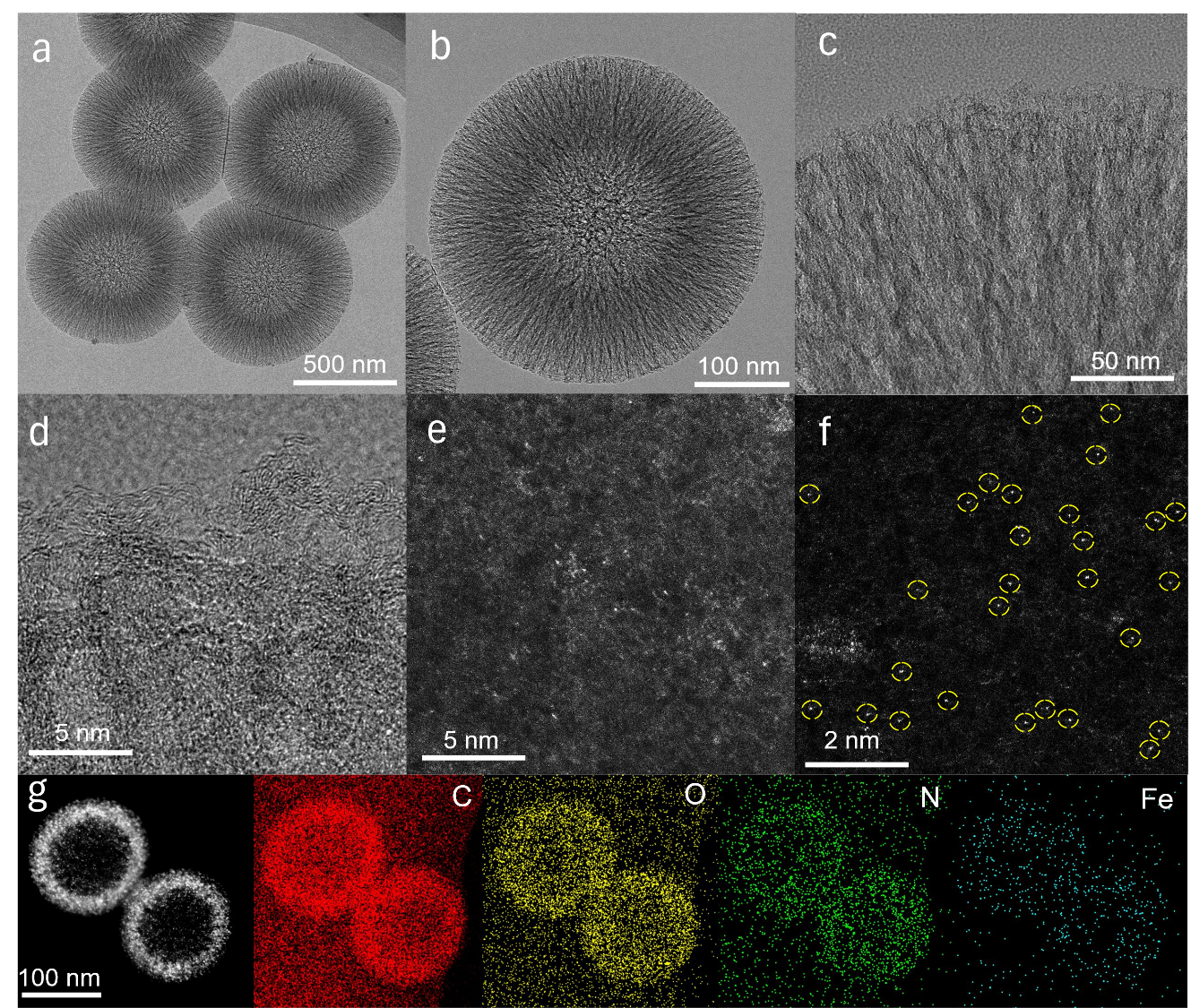


Fig. S2. a-d) HRTEM images of the as-obtained FeNC-MCS; e) HAADF-STEM image of FeNC-MCS; f) Zoomed-in HAADF-STEM image indicates the dispersion of metal atoms sites where bright dots were marked with yellow dashed circles; and g) HAADF-STEM image and EDS mapping of Fe, N, O, and C elements over FeNC-MCS.


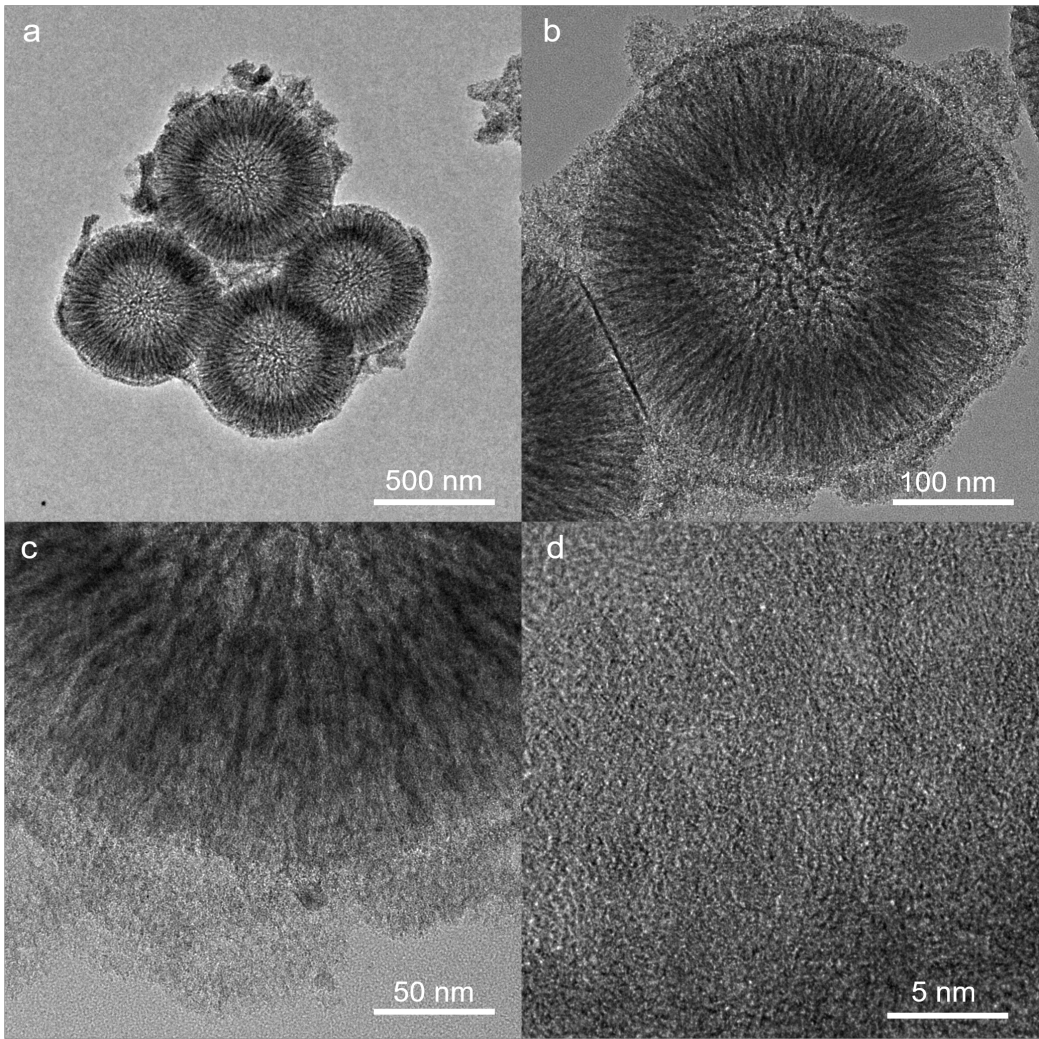


Fig. S3. HRTEM images of FeNC@MgNC-DMCS(1).


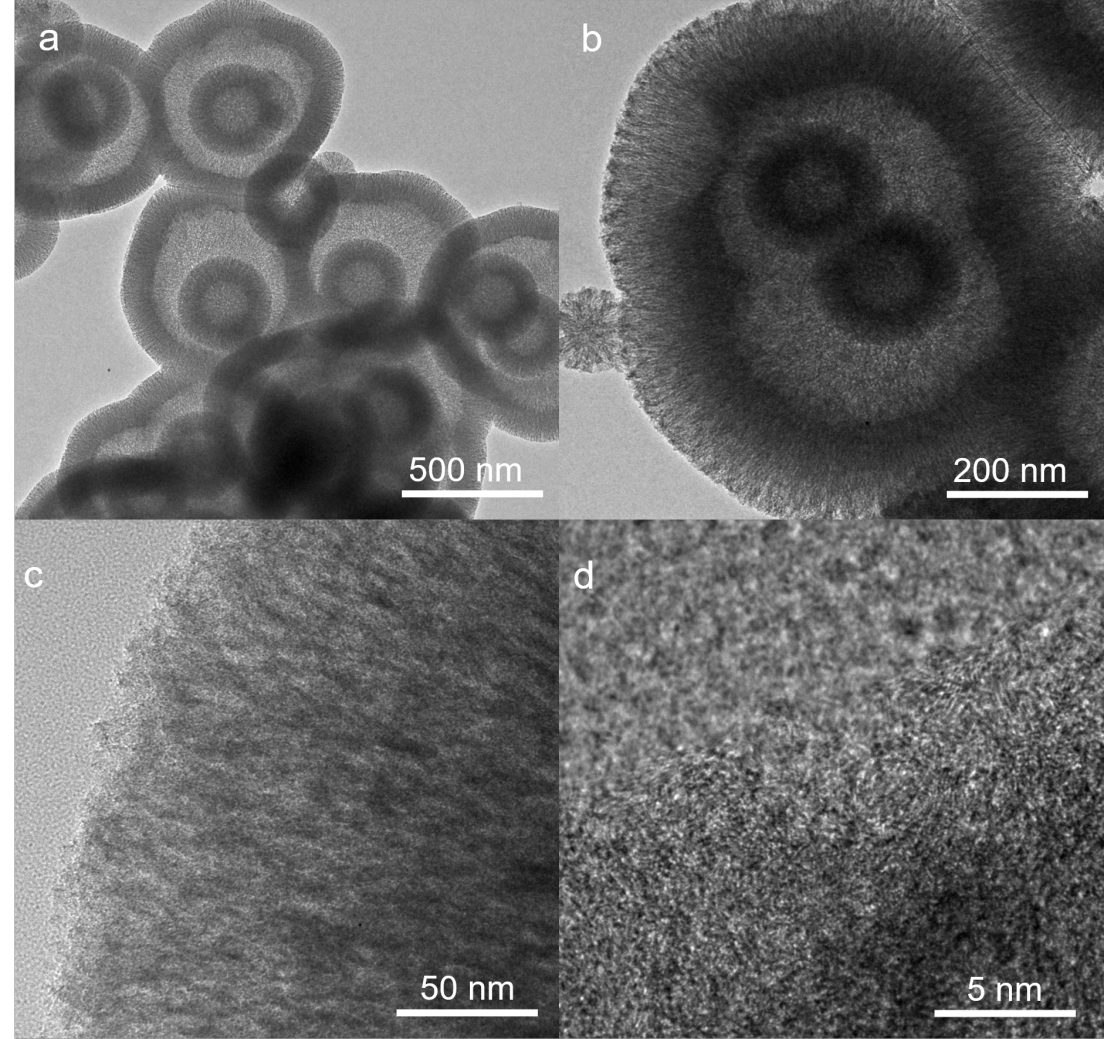


Fig. S4. HRTEM images of FeNC@MgNC-DMCS(3).


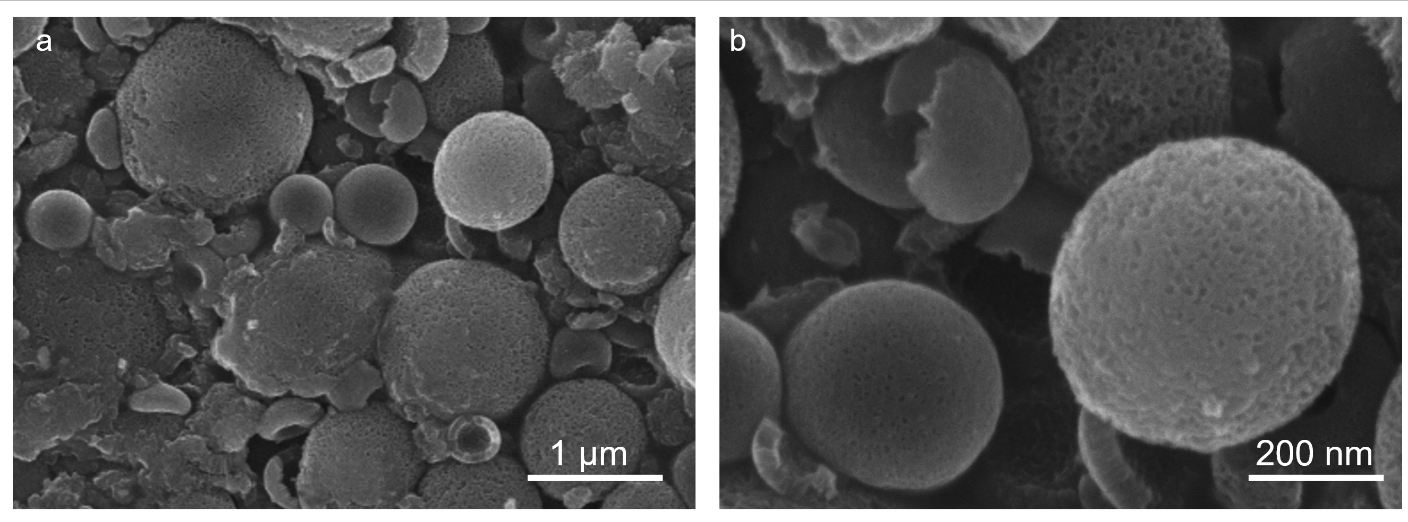


Fig. S5. SEM images of FeNC@MgNC-DMCS.


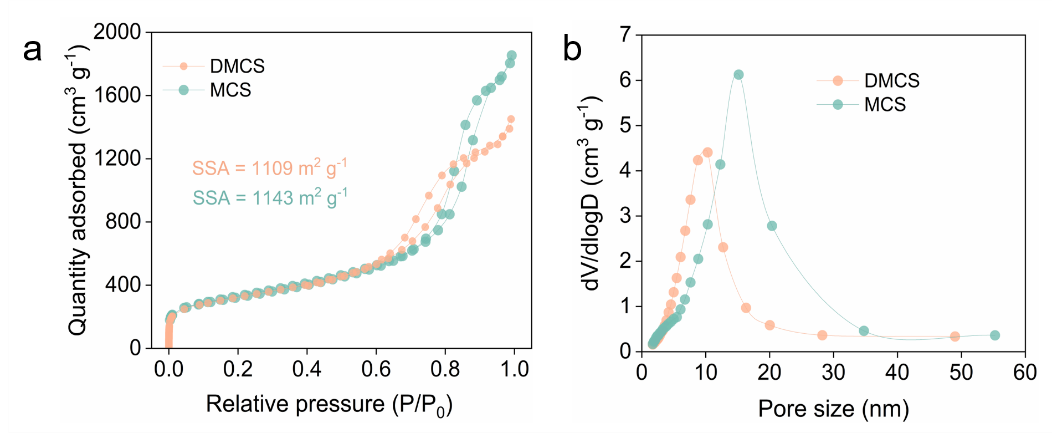


Fig. S6. a) Nitrogen adsorption-desorption isotherms, and b) pore size distribution curves of DMCS, and MCS.


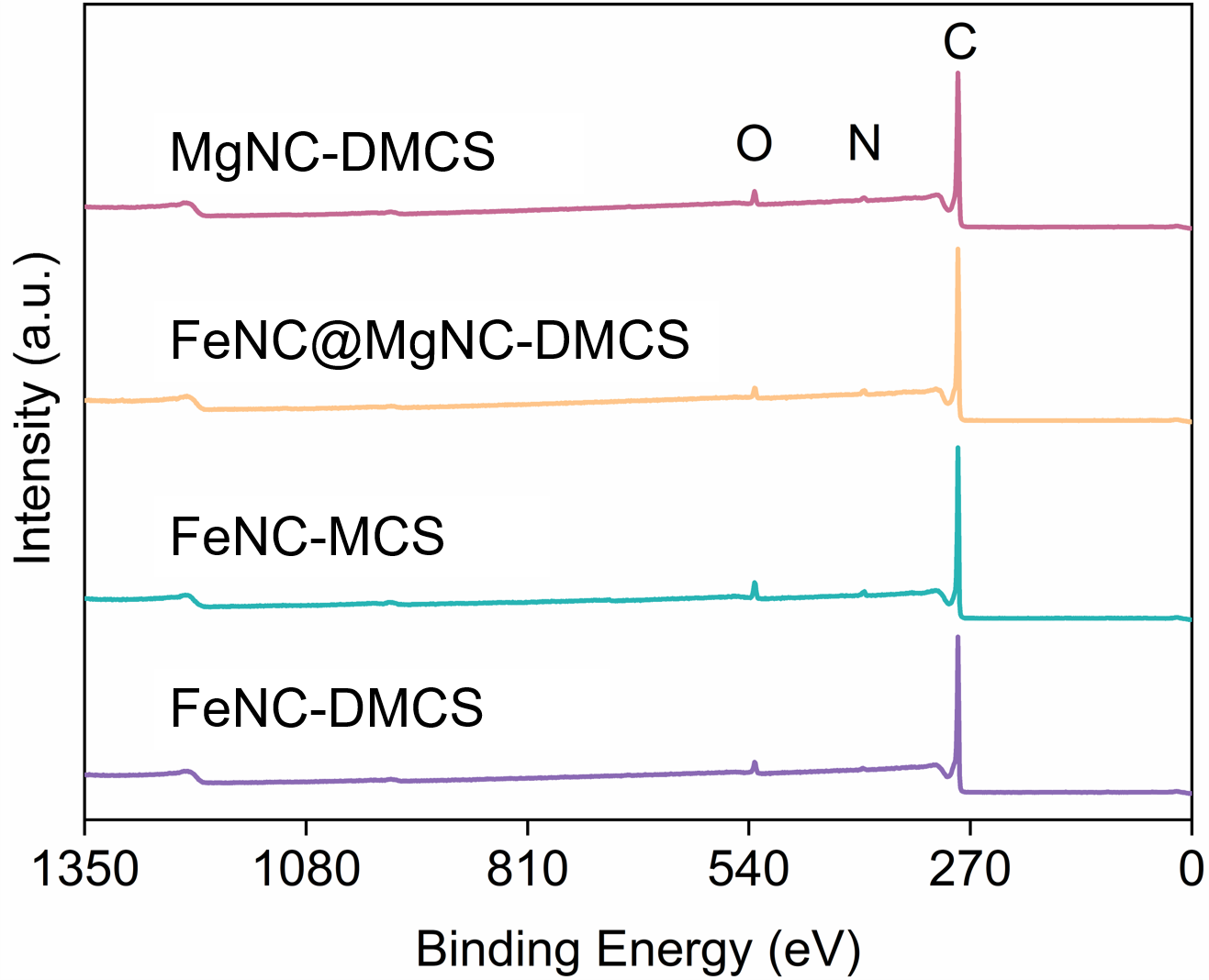


Fig. S7. XPS survey spectra of FeNC-MCS, FeNC-DMCS, MgNC-DMCS, and FeNC@MgNC-DMCS.


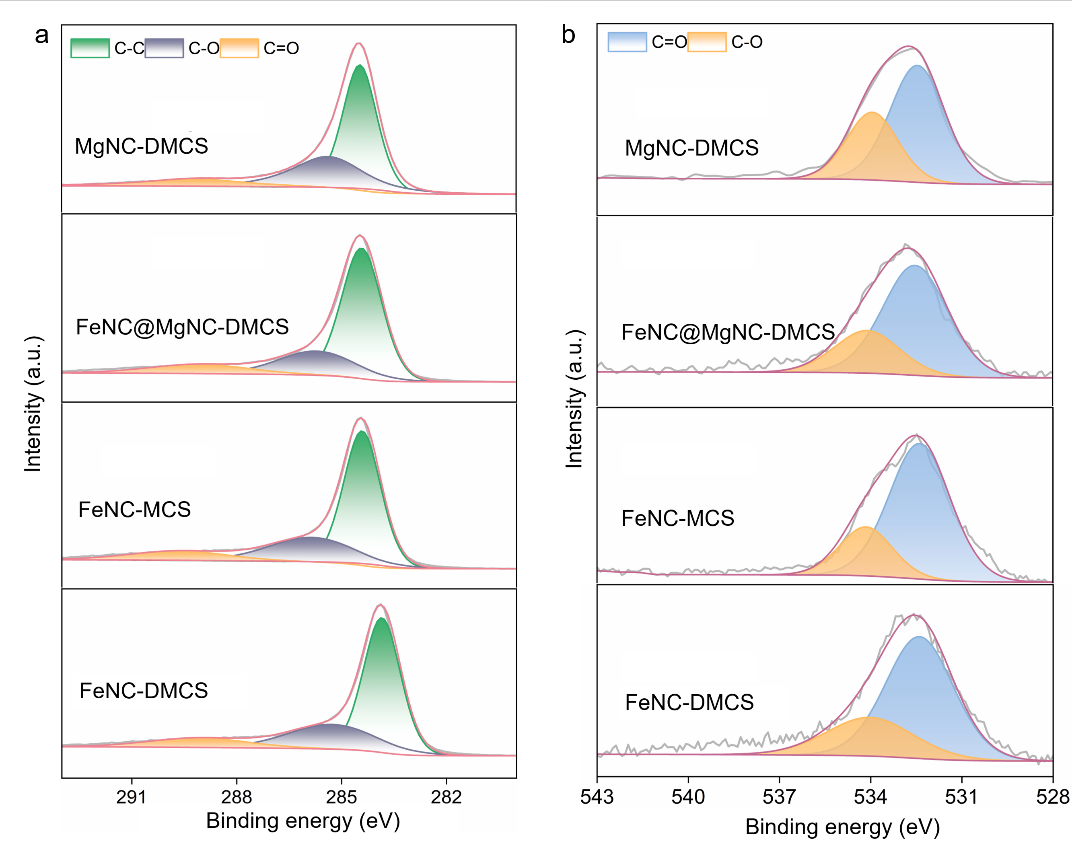


Fig. S8. High-resolution XPS spectra of a) C 1s and b) O 1s for FeNC-MCS, FeNC-DMCS, MgNC-DMCS, and FeNC@MgNC-DMCS.


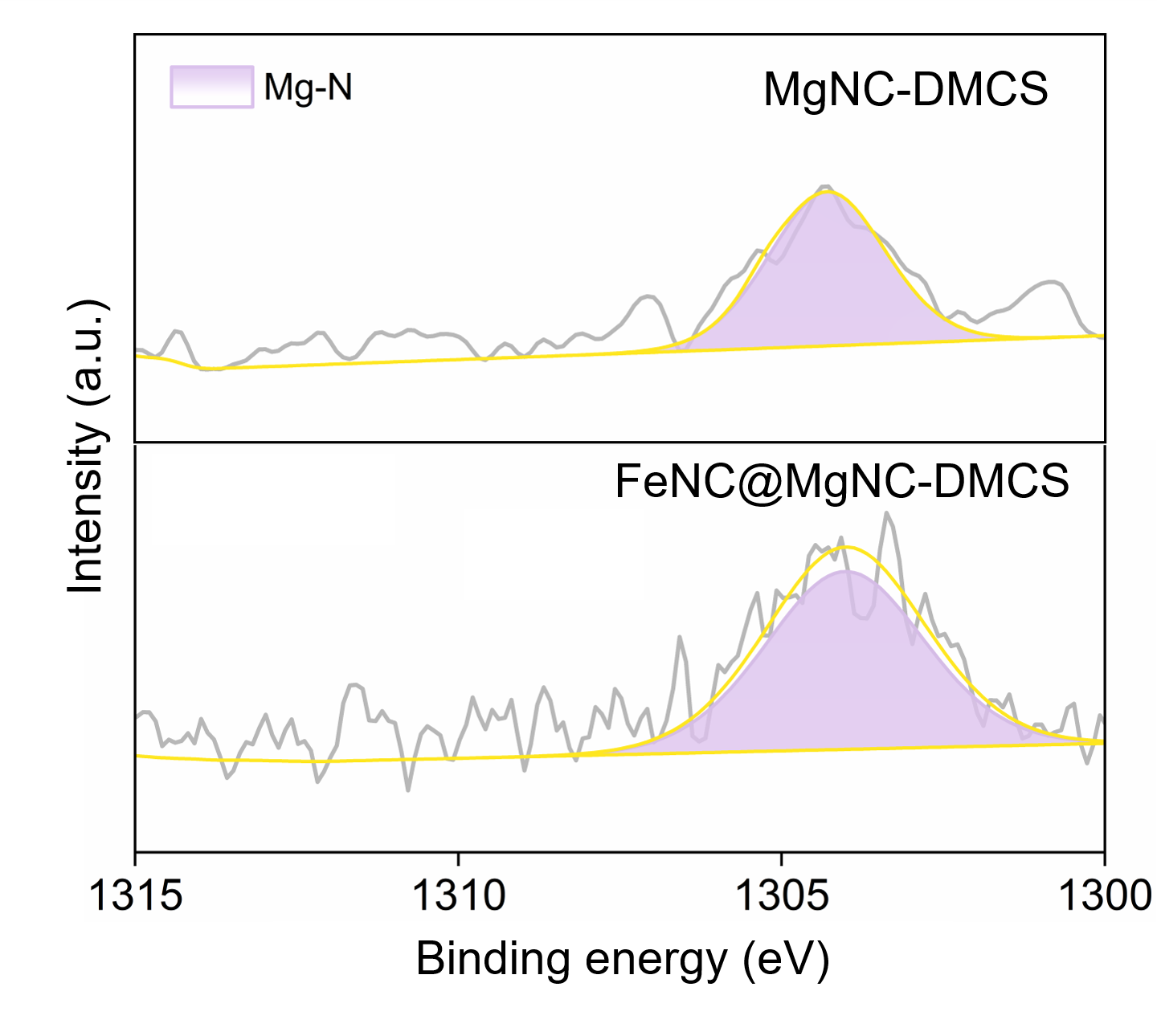


Fig. S9. High-resolution XPS spectra of Mg 1s for MgNC-DMCS, and FeNC@MgNC-DMCS.


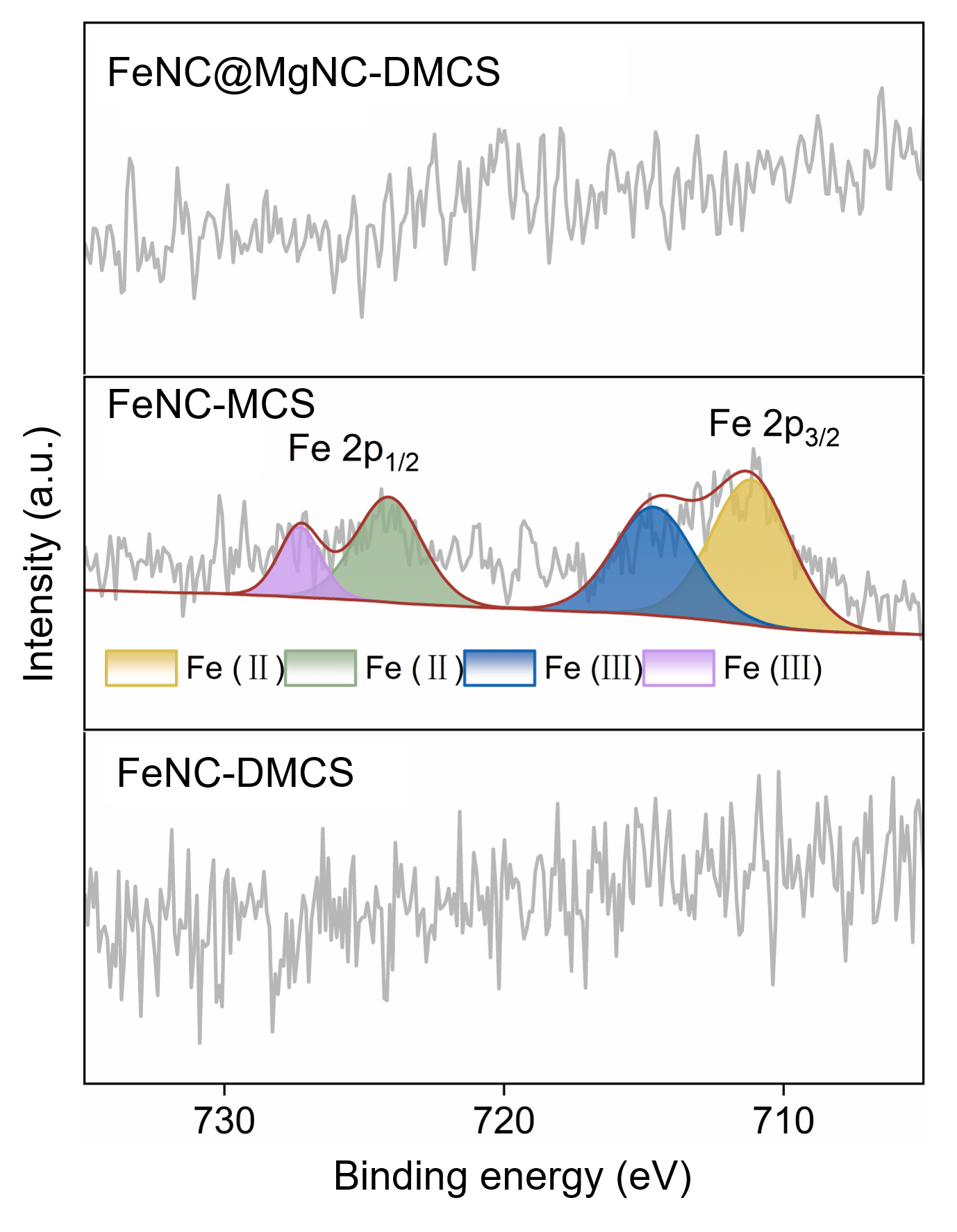


Fig. S10. High-resolution XPS spectra of Fe 2p for FeNC-MCS, FeNC-DMCS, and FeNC@MgNC-DMCS.


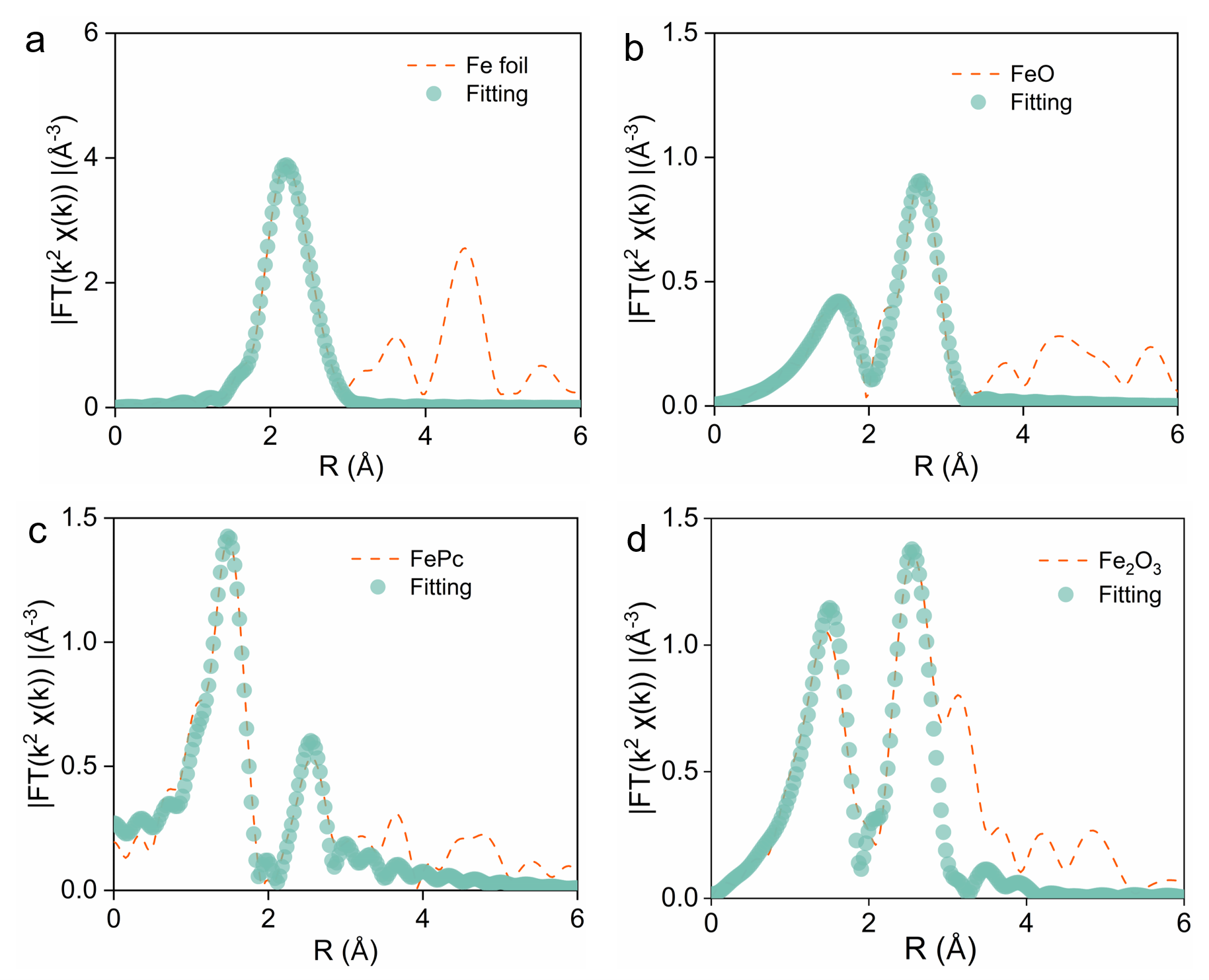


Fig. S11. EXAFS fittings of Fe foil (a), FeO (b), FePc (c), and Fe_2_O_3_ (d) in the R space at the Fe K-edge.


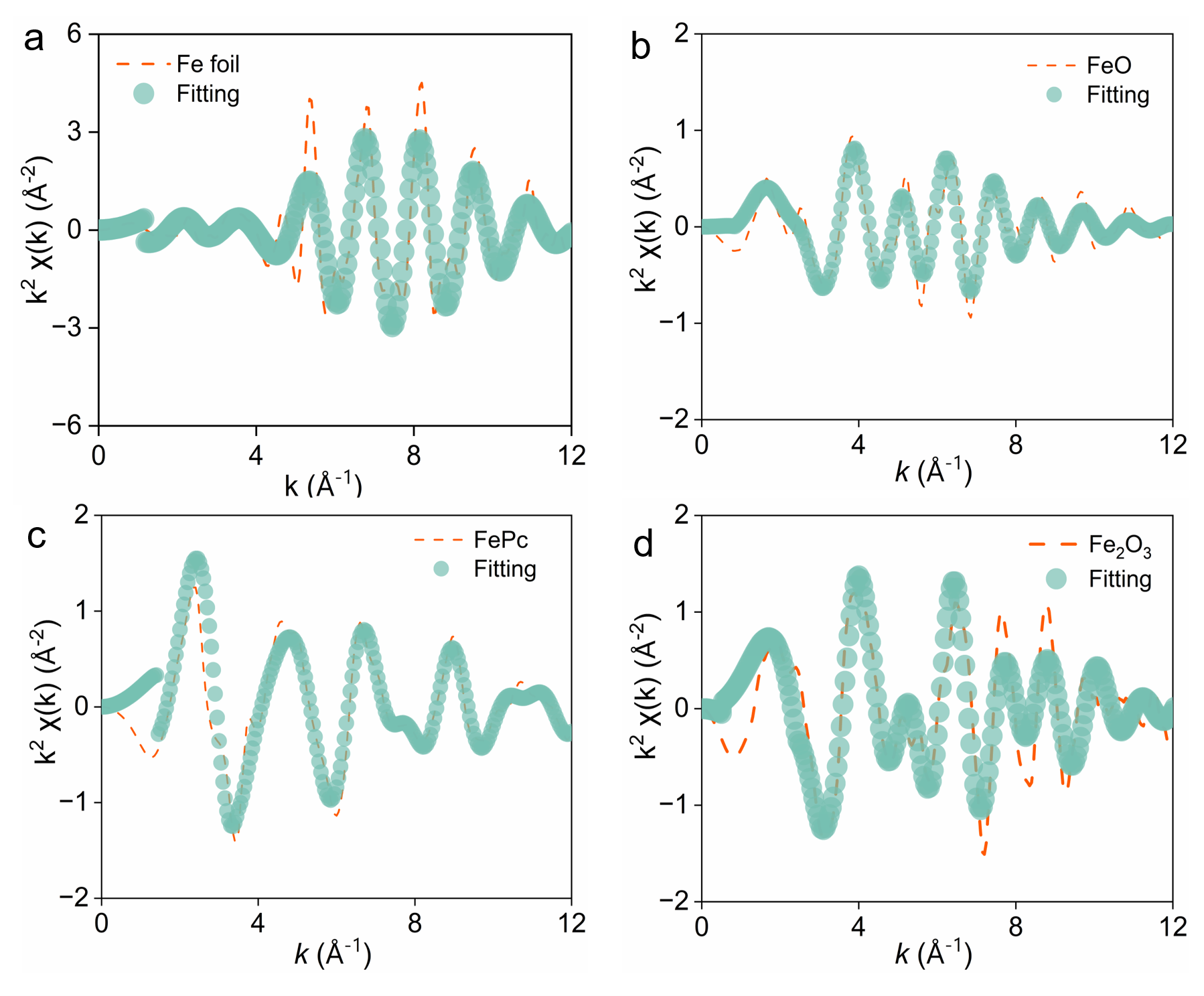


Fig. S12. EXAFS fittings of Fe foil (a), FeO (b), FePc (c), and Fe_2_O_3_ (d) in the K space at the Fe K-edge.


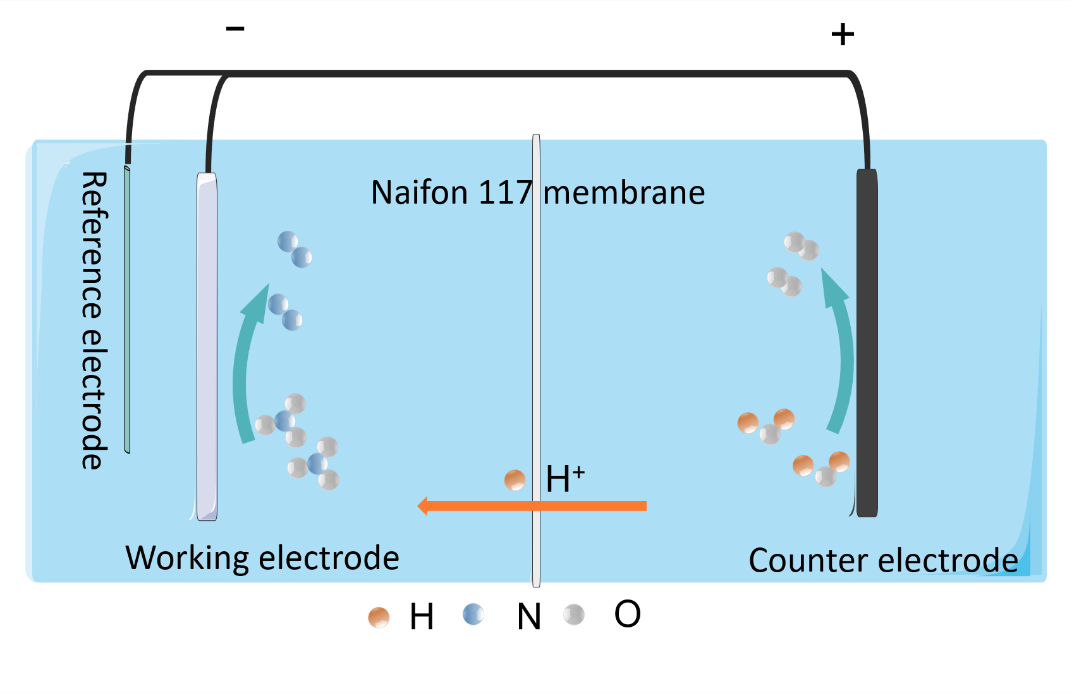


Fig. S13. The schematic illustration of electrochemically double-cell reactor.


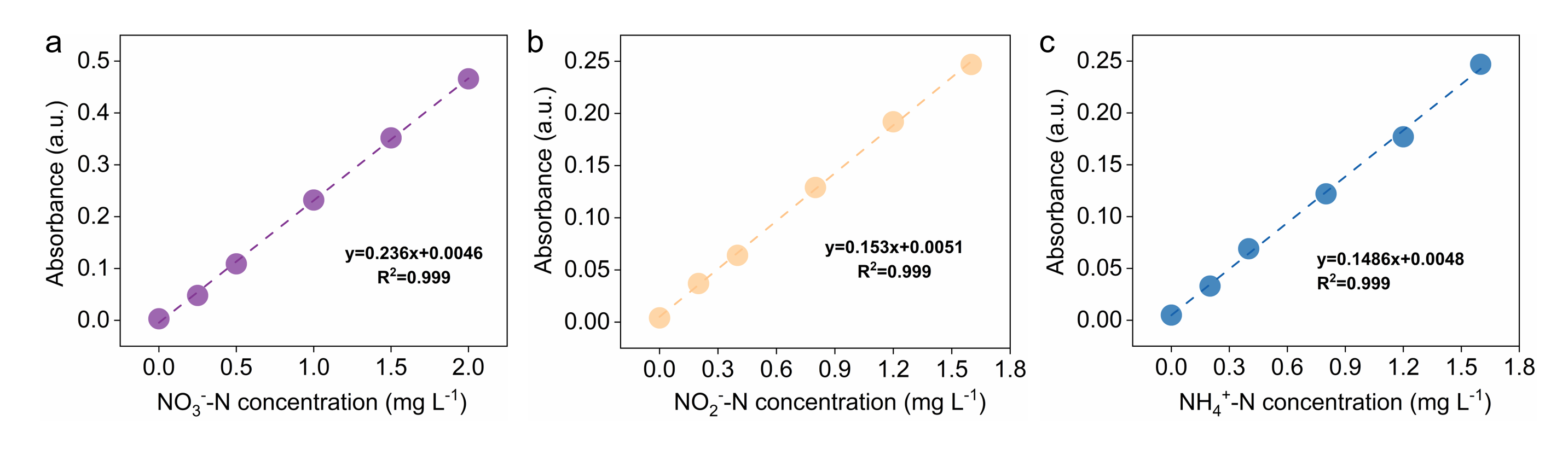


Fig. S14. The concentration-absorbance standard curves of a) NO_3_^–^, b) NO_2_^–^ and c) NH_4_^+^.


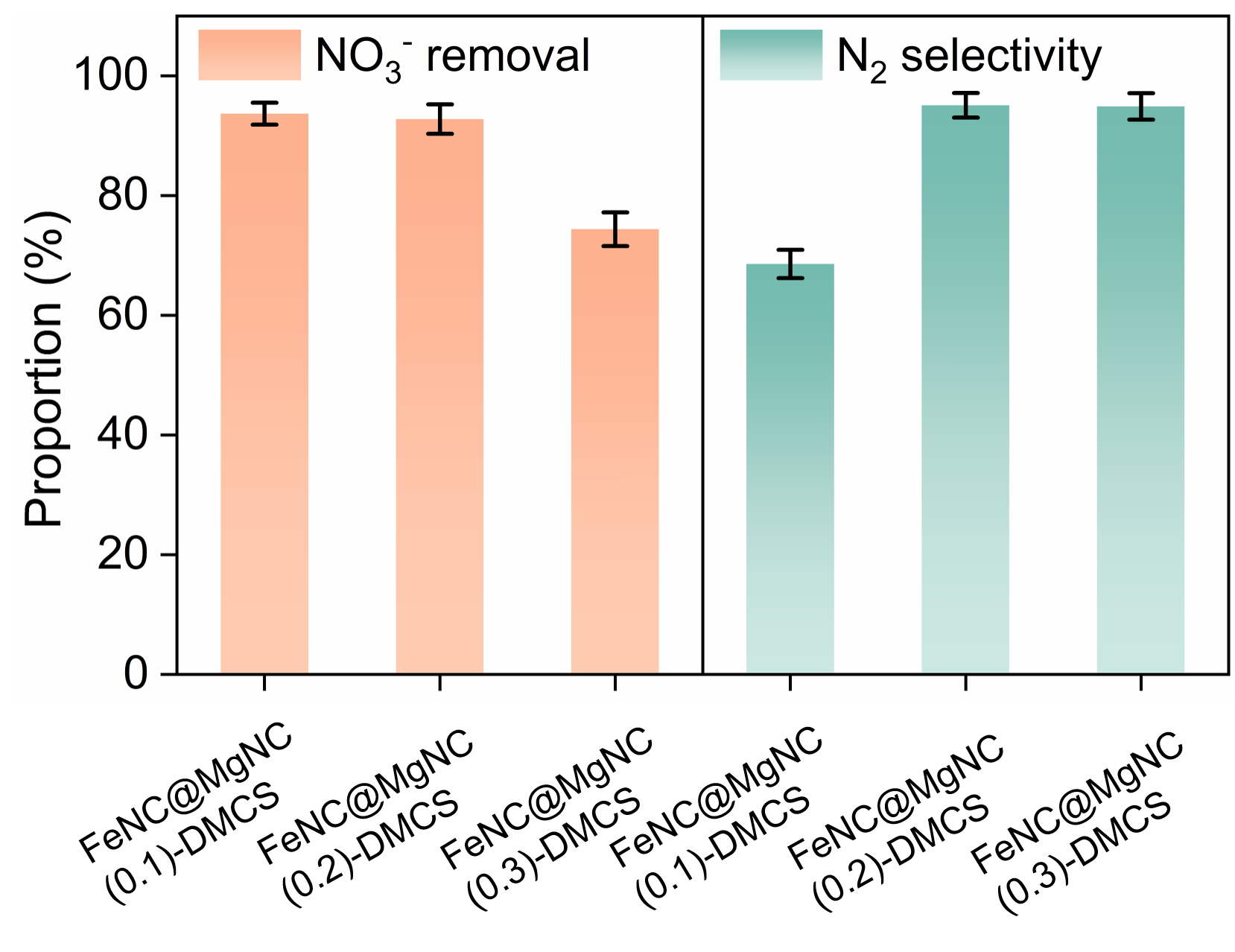


Fig. S15. Effects oflevel of addition Mg in FeNC@MgNC-DMCS on the NO_3_^–^ removal rate and N_2_ selectivity.





Fig. S16. LSV curves of MCS, and DMCS.





Fig. S17. LSV curves of the FeNC@MgNC-DMCS 0.05 M Na_2_SO_4_ and 0.05 M Na_2_SO_4_ with100 mg L^−1^ NO_3_^−^.





Fig. S18. Chronoamperometric curves of FeNC@MgNC-DMCS 0.05 M Na_2_SO_4_ and 0.05 M Na_2_SO_4_ with100 mg L^−1^ NO_3_^−^ at −1.3 V (vs. SCE) for 12 h.


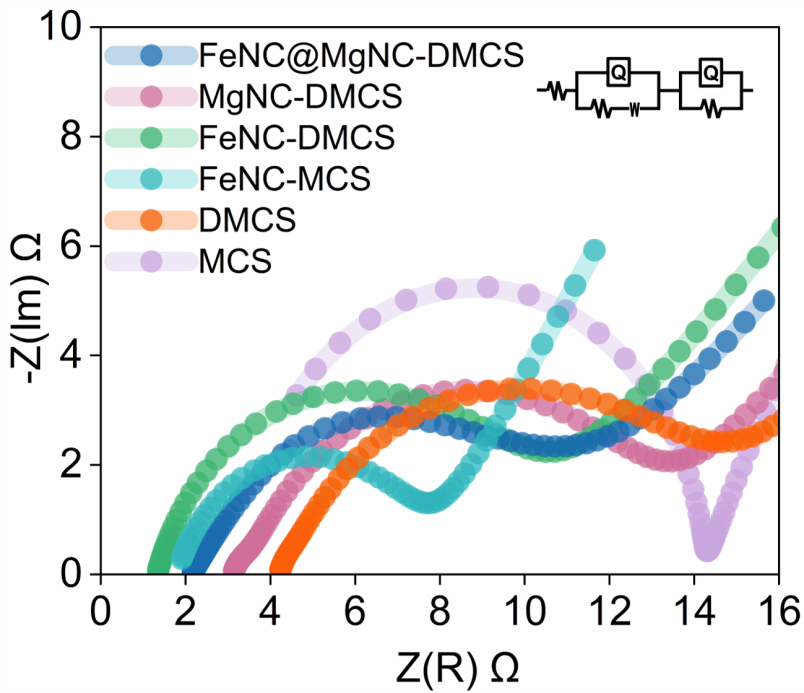


Fig. S19. EIS plots of MCS, DMCS, FeNC-MCS, FeNC-DMCS, MgNC-DMCS, and FeNC@MgNC-DMCS (inset: equivalent circuit diagram of EIS tests).


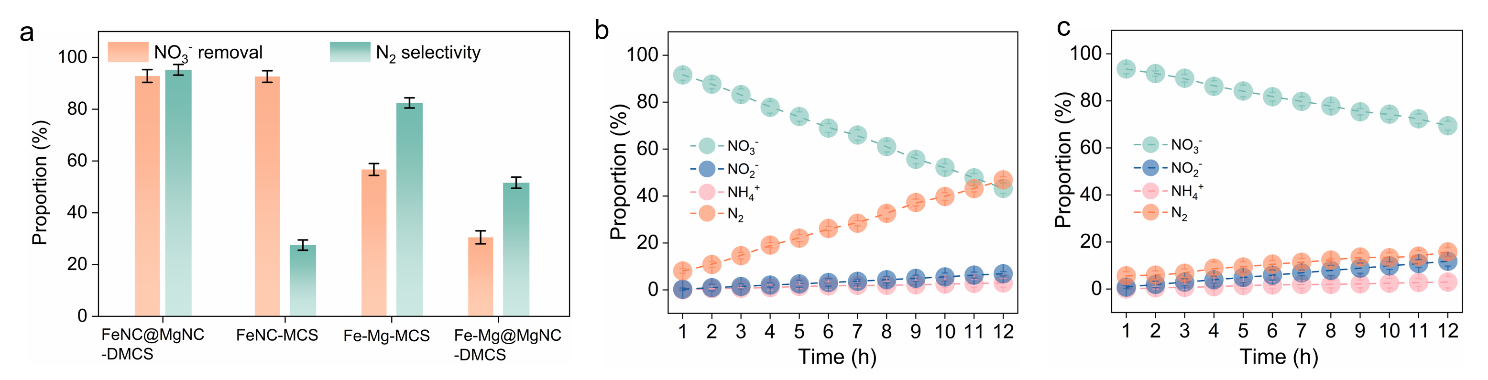


Fig. S20. a) NO_3_^−^ removal rate and N_2_ selectivity of FeNC@MgNC-DMCS, FeNC-MCS, Fe-Mg-MCS, and Fe-Mg@MgNC-DMCS for 12 h; b, c) Concentration of NO_3_^−^, NO_2_^−^, NH_4_^+^, and N_2_ as a function of time during the NO_3_^−^ reduction over Fe-Mg-MCS (b), and Fe-Mg@MgNC-DMCS (c).

*Note: To verify the influence of Mg sites in both inner and outer shells of the double-shell structure on ECDN activity, Fe-Mg@MgNC-DMCS (0.26wt% Fe, 1.2wt% Mg) was synthesized using Fe-Mg-MCS (1.83wt% Fe, 1.32wt% Mg) as the precursor. Fe-Mg@MgNC-DMCS showed insufficient nitrate reduction kinetics, with NO_3_^−^ removal rate and N_2_ selectivity at 30.5% and 51.6%, respectively (Fig. S18). These results suggested that the enhanced catalytic performance of FeNC@MgNC-DMCS primarily stems from the spatial separation of Fe and Mg active sites through the engineered double-shell carbon architecture. EDS elemental mapping confirmed confirmed that the Mg sites of FeNC@MgNC-DMCS were uniformly distributed as a whole without obvious aggregation in the inner shell.


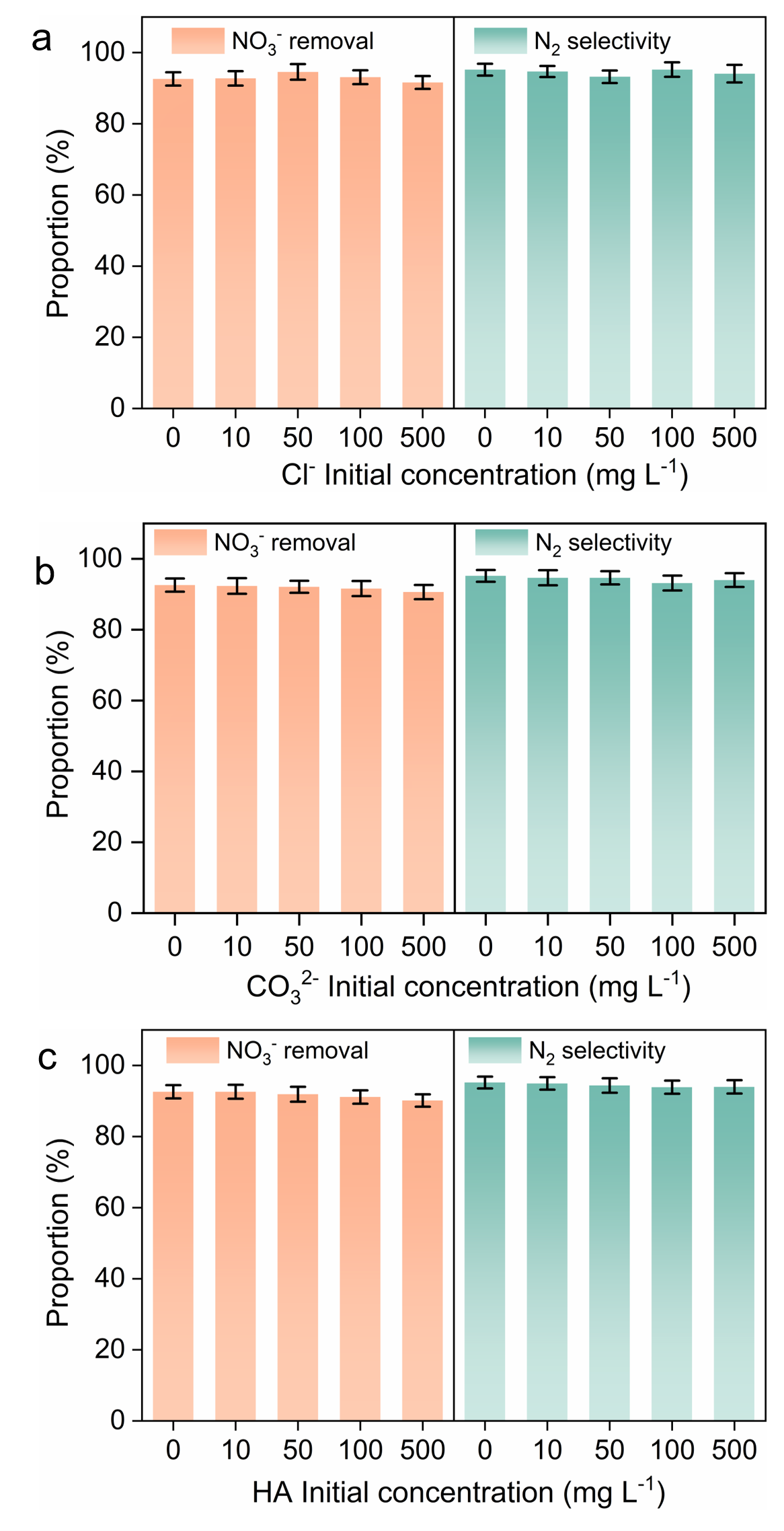


Fig. S21. ECDN activity over FeNC@MgNC-DMCS at different initial concentrations of Cl^−^, CO_3_^2−^ and HA (Humic acid).


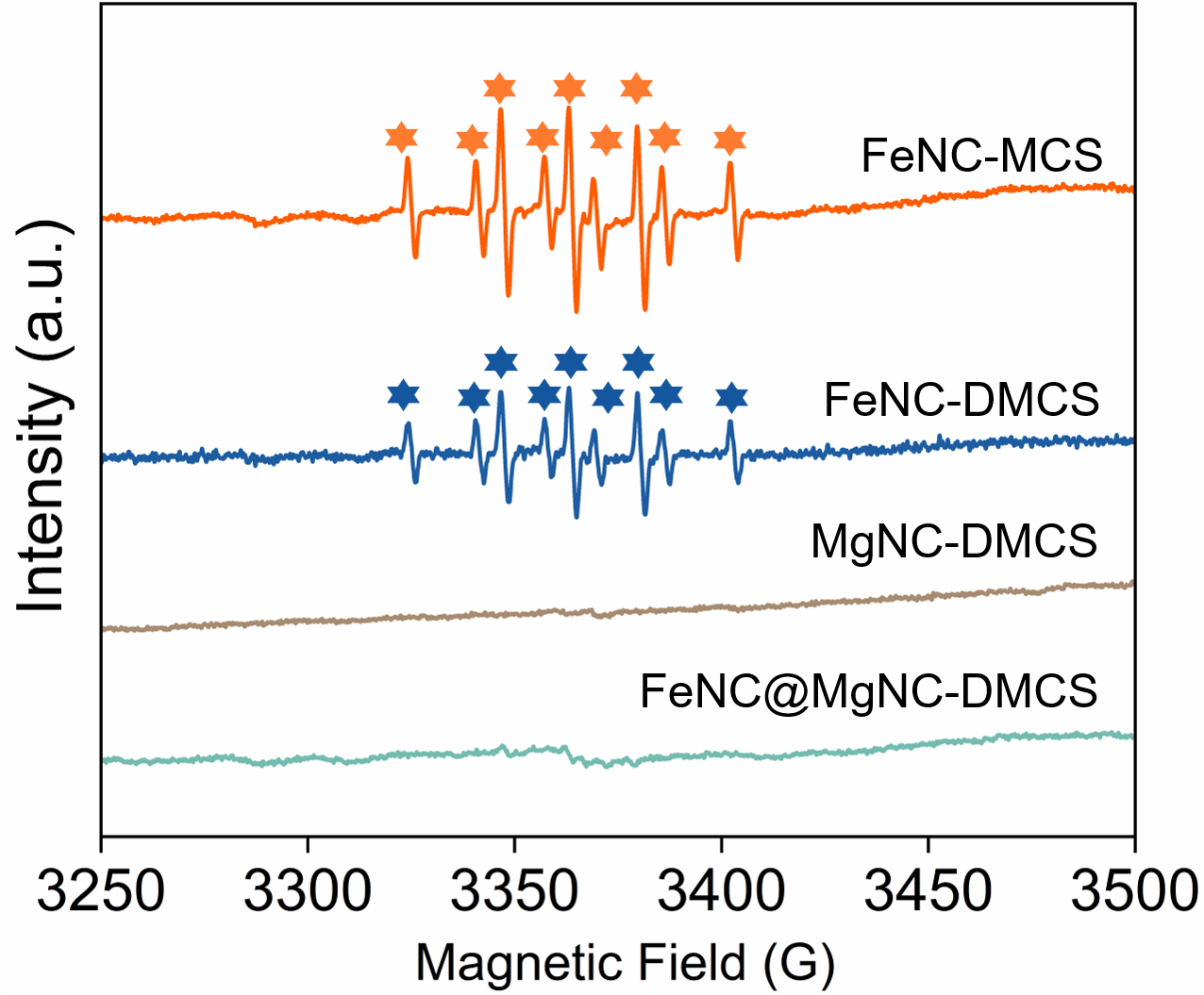


Fig. S22. EPR spectras of the FeNC-MCS, FeNC-DMCS, MgNC-DMCS, and FeNC@MgNC-DMCS using DMPO (50 mM) as the spin-trapping agent in an electrolyte of 0.05 M Na_2_SO_4_;





Fig. S23. FE for N_2_ of FeNC@MgNC-DMCS at varied potentials.


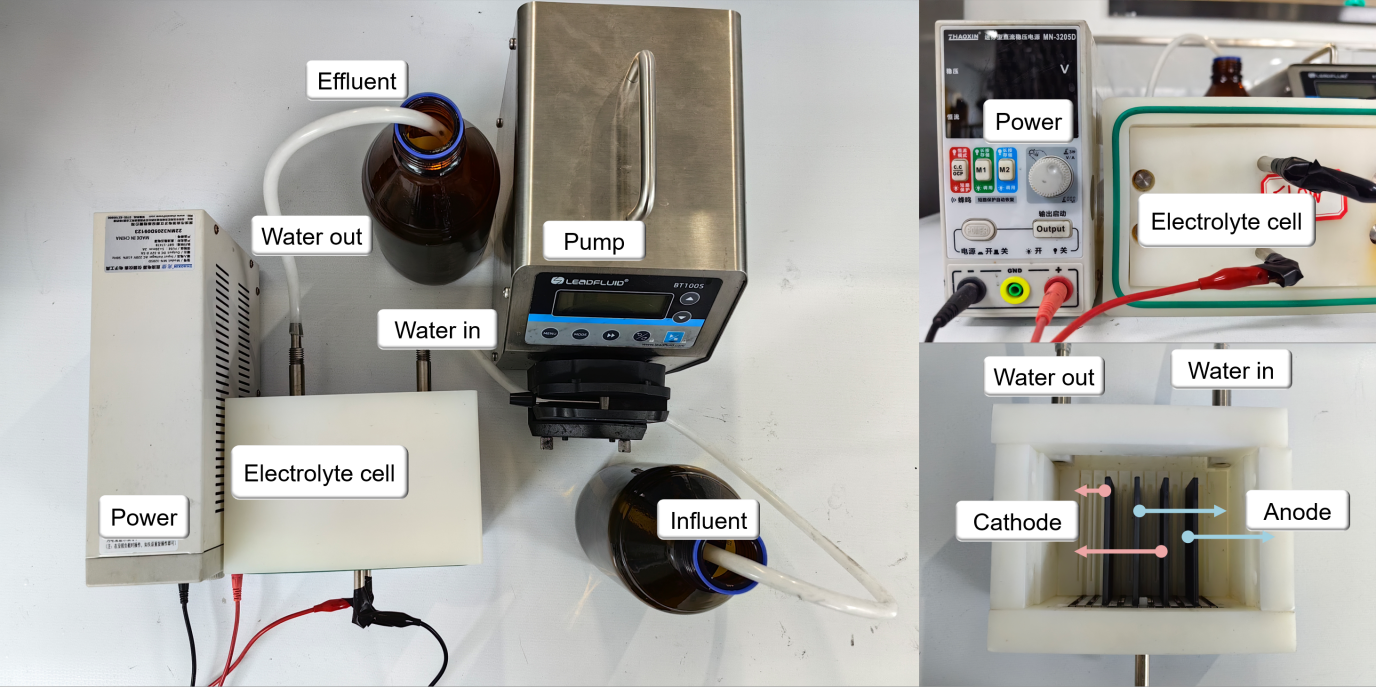


Fig. S24. Photographs of theflow cell of FeNC@MgNC-DMCS.





Fig. S25. LSV curves of the initial and after cycling tests of FeNC@MgNC-DMCS.


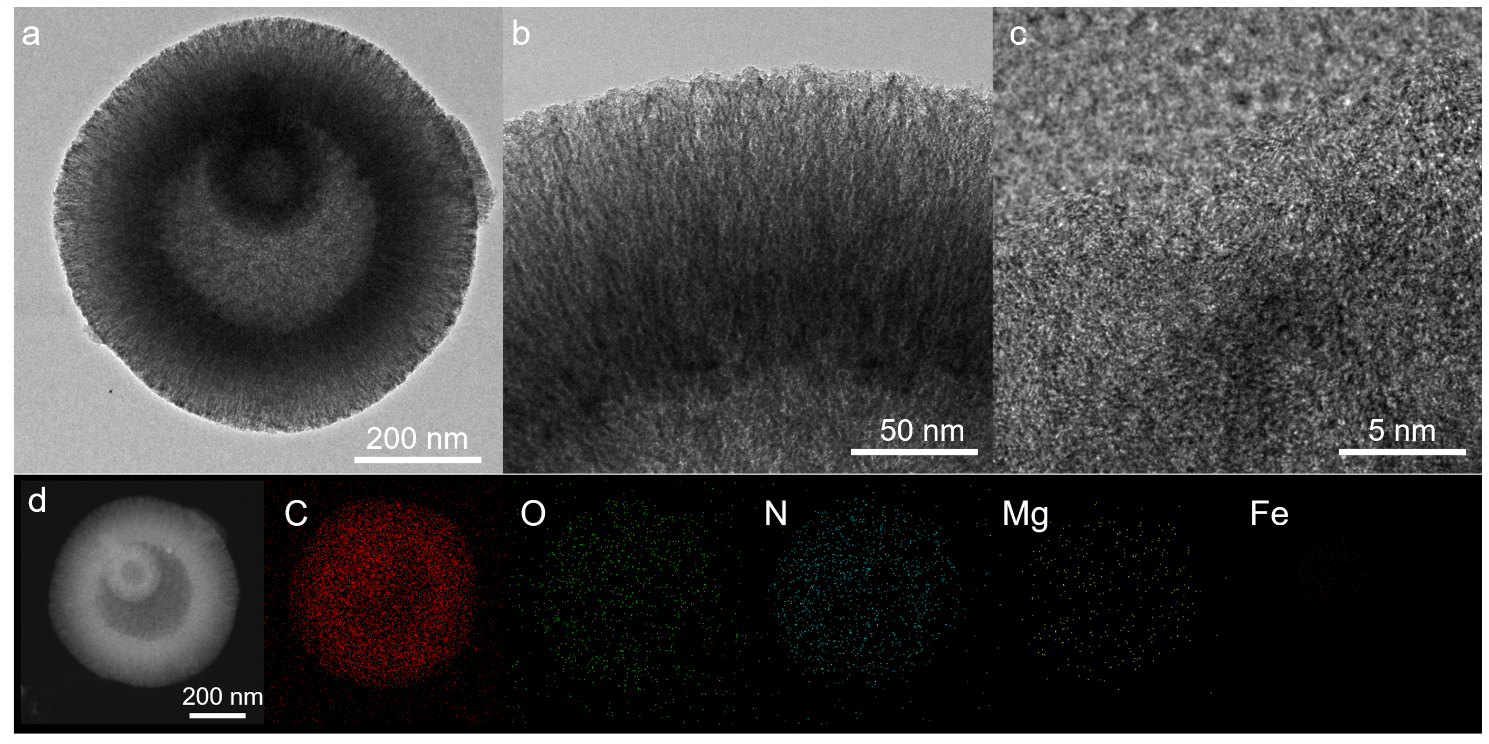


Fig. S26. a) HRTEM images of FeNC@MgNC-DMCS after the catalytic reaction; b) EDS mapping of Mg, Fe, N, O, and C elements of FeNC@MgNC-DMCS after the catalytic reaction.





Fig. S27. XRD images of FeNC@MgNC-DMCS after the catalytic reaction.


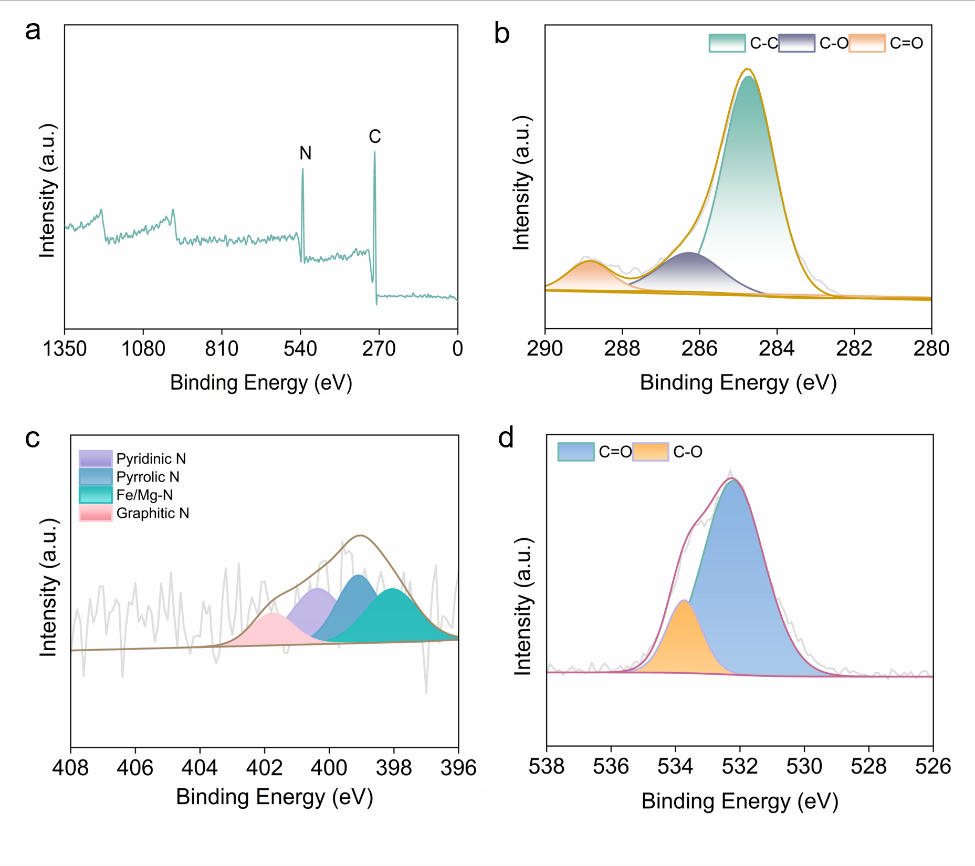


Fig. S28. a) XPS images of FeNC@MgNC-DMCS after the catalytic reaction; b) C 1s, c) N 1s, and d) O 1s.

3. Supplementary Tables

**Table S1** Fe contents of the samples measured by ICP-MS.

| Sample | Fe contents (wt%) | Mg contents (wt%) |
| --- | --- | --- |
| FeNC-MCS | 3.16 | / |
| FeNC-DMCS | 0.42 | / |
| MgNC-DMCS | / | 1.32 |
| FeNC@MgNC(0.1)-DMCS | 0.43 | 0.46 |
| FeNC@MgNC(0.2)-DMCS | 0.43 | 0.97 |
| FeNC@MgNC(0.3)-DMCS | 0.42 | 1.52 |

**Table S2** EXAFS structure parameters of FeNC@MgNC.

| Sample | Shell | CN | R (Å) | σ^2^ (10^-3^Å^2^) | ΔE_0_ (eV) | R factor |
| --- | --- | --- | --- | --- | --- | --- |
| Fe foil | Fe−Fe | 12(fixed) | 2.49±0.01 | 4.1±1.6 | 3.1±5.2 | 0.002 |
| FePc | Fe−N | 3.9±0.3 | 1.92±0.01 | 1.1±1.6 | 1.2±4.1 | 0.009 |
|  | Fe−C | 7.5±0.4 | 2.94±0.01 | 3.2±6.1 |  |  |
|  | Fe−Fe | 13.9±2.1 | 3.12±0.01 | 1.8±2.8 |  |  |
| FeO | Fe−O | 5.8±0.5 | 2.17±0.01 | 5.2±2.8 | −1.9±4.2 | 0.01 |
|  | Fe−Fe | 11.3±0.9 | 3.06±0.01 | 2.1±1.8 | 2.3±3.7 |  |
| Fe_2_O_3_ | Fe−O | 2.8±0.3 | 2.01±0.01 | 3.7±1.9 | −3.6±5.7 | 0.014 |
|  | Fe−Fe | 11.5±0.9 | 2.98±0.01 |  |  |  |
| FeNC@MgNC | Fe−N | 4.2±0.4 | 2.01±0.01 | 5.0±4.8 | −2.7±2.1 | 0.006 |

Data ranges: 3.0 ≤ k ≤ 12.0 Å^−1^, 1.0 ≤ R ≤ 3.0 Å. R: bond distance; σ^2^: Debye-Waller factors; R factor: goodness of fit. S0^2^ is the amplitude reduction factor (S_0_^2^ =0.82 was obtained by Fe foil fitting and applied for the other samples fitting).

**Table S3** A comparison of the NO_3_^−^ reduction activities for reported catalysts.

| Materials | Electrolyte conditions | NO_3_^−^ removal (%) | N_2_ Selectivity (%) | Reaction time (h) | Ref. |
| --- | --- | --- | --- | --- | --- |
| Fe(20%)@N-C | 50 mg L^−1^ NO_3_^−^, 50 mM Na_2_SO_4_ | 83 | 25 | 24 | [4] |
| Pd-Cu/PNC | 100 mg L^−1^ NO_3_^−^, 0.1 M Na_2_SO_4_ | 97.1 | 83 | 24 | [5] |
| Ad Pd-Cu/NF | 200 mg L^−1^ NO_3_^−^ | 37 | 92 | 2 | [6] |
| Cu-COP@CNTs | 0.1 M NaNO_3_, 0.1 M Na_2_SO_4_ | 66 | 93 | 12 | [7] |
| CuPd@N-OMC | 100 mg L^−1^ NO_3_^−^, 0.1M Na_2_SO_4_ | 86 | 84 | 24 | [8] |
| Cu_28_Pd_72_ NWs | 22.5 mg L^−1^ NO_3_^−^, 50 mM Na_2_SO_4_ | ~100 | 75.9 | 4 | [9] |
| Meso-Fe-N-C | 50 mg L^−1^ NO_3_^−^, 0.1 M Na_2_SO_4_ | 77 | 85 | 24 | [10] |
| Sn_2_/NCB | 50 mg L^−1^ NaNO_3_, 50 mM Na_2_SO_4_ | 401.6 mg-N h^−1^ m^−2^ | 86 | / | [11] |
| CNT-EM | 1 M Na_2_SO_4_ or Na_2_SO_4_ + KNO_3_ | 60.8% | 72 | / | [12] |
| RLFe2N@NC | 0.02 M NaCl, 0.02 M Na_2_SO_4_, 100 mg L^−1^ NO_3_^−^ | 86 | 97 | 24 | [13] |
| FeNC@MgNC-DMCS | 100 mg L^−1^ NO_3_^−^, 0.05 M Na_2_SO_4_ | 92.8 | 95.2 | 12 | This work |

**Table S4** Characteristics of ROC samples.

| Parameter | Value |
| --- | --- |
| COD (mg L^−1^) | 85.5 ± 7.5 |
| pH | 7.7 ± 0.5 |
| NO_3_^−^ (mg L^−1^) | 103.6 ± 5.7 |
| NO_2_^−^ (mg L^−1^) | 23.1 ± 4.1 |
| SO_4_^2+^ (mg L^−1^) | 2471.5 ± 85.3 |
| Na^+^ (mg L^−1^) | 525.6 ± 15.6 |
| K^+^ (mg L^−1^) | 131.2 ± 10.4 |
| Ca^2+^ (mg L^−1^) | 415.7 ± 11.8 |
| Mg^2+^ (mg L^−1^) | 86.2 ± 10.2 |

References

[1] S. Zhang, J. Wu, M. Zheng, X. Jin, Z. Shen, Z. Li, Y. Wang, Q. Wang, X. Wang, H. Wei, J. Zhang, P. Wang, S. Zhang, L. Yu, L. Dong, Q. Zhu, H. Zhang, J. Lu, Fe/Cu diatomic catalysts for electrochemical nitrate reduction to ammonia, Nat. Commun., 14 (2023) 3634.

[2] H. Li, S. Li, R. Guan, Z. Jin, D. Xiao, Y. Guo, P. Li, Modulating the surface concentration and lifetime of active hydrogen in Cu-based layered double hydroxides for electrocatalytic nitrate reduction to ammonia, ACS Catal., 14 (2024) 12042-12050.

[3] J. Leverett, T. Tran‐Phu, J.A. Yuwono, P. Kumar, C. Kim, Q. Zhai, C. Han, J. Qu, J. Cairney, A.N. Simonov, R.K. Hocking, L. Dai, R. Daiyan, R. Amal, Tuning the coordination structure of Cu-N-C single atom catalysts for simultaneous electrochemical reduction of CO_2_ and NO_3_^–^ to urea, Adv. Energy Mater., 12 (2022) 2201500.

[4] W. Duan, G. Li, Z. Lei, T. Zhu, Y. Xue, C. Wei, C. Feng, Highly active and durable carbon electrocatalyst for nitrate reduction reaction, Water Res., 161 (2019) 126-135.

[5] T. Gu, W. Teng, N. Bai, Z. Chen, J. Fan, W.-x. Zhang, D. Zhao, Nano-spatially confined Pd–Cu bimetals in porous N-doped carbon as an electrocatalyst for selective denitrification, J. Mater. Chem. A., 8 (2020) 9545-9553.

[6] X. Bian, F. Shi, J. Li, J. Liang, C. Bao, H. Zhang, J. Jia, K. Li, Highly selective electrocatalytic reduction of nitrate to nitrogen in a chloride ion-free system by promoting kinetic mass transfer of intermediate products in a novel Pd-Cu adsorption confined cathode, J. Environ. Manage., 324 (2022) 116405.

[7] Z. Yin, J. Liu, L. Jiang, J. Chu, T. Yang, A. Kong, Semi-enclosed Cu nanoparticles with porous nitrogen-doped carbon shells for efficient and tolerant nitrate electroreduction in neutral condition, Electrochim. Acta., 404 (2022) 139585.

[8] H. Xu, H. Xu, Z. Chen, X. Ran, J. Fan, W. Luo, Z. Bian, W.-x. Zhang, J. Yang, Bimetallic pdcu nanocrystals immobilized by nitrogen-containing ordered mesoporous carbon for electrocatalytic denitrification, ACS Appl. Mater. Interfaces, 11 (2019) 3861-3868.

[9] C. Fu, S. Shu, L. Hu, Z. Liu, Z. Yin, X. Lv, S. Zhang, G. Jiang, Electrocatalytic nitrate reduction on bimetallic palladium-copper nanowires: Key surface structure for selective dinitrogen formation, Chem. Eng. J., 435 (2022) 134969.

[10] J. Fan, Y. Chen, X. Chen, Z. Wu, W. Teng, W.-x. Zhang, Atomically dispersed iron enables high-efficiency electrocatalytic conversion of nitrate to dinitrogen on a N-coordinated mesoporous carbon architecture, Appl. Catal. B-Environ., 320 (2023) 121983.

[11] X. Wu, X. Wang, Y. Wu, H. Xu, Z. Li, R. Hong, K. Rigby, Z. Wu, J.-H. Kim, Bilayer electrified-membrane with pair-atom tin catalysts for near-complete conversion of low concentration nitrate to dinitrogen, Nat. Commun., 16 (2025) 1122.

[12] Y. Fan, X. Wang, C. Butler, A. Kankam, A. Belgada, J. Simon, Y. Gao, E. Chen, L.R. Winter, Highly efficient metal-free nitrate reduction enabled by electrified membrane filtration, Nat. Water, 2 (2024) 684-696.

[13] H. Luo, S. Li, Z. Wu, Y. Liu, W. Luo, W. Li, D. Zhang, J. Chen, J. Yang, Modulating the active hydrogen adsorption on Fe-N interface for boosted electrocatalytic nitrate reduction with ultra-long stability, Adv. Mater., 35 (2023) e2304695.
